# Functional Heterodimerization between the G Protein-Coupled Receptor GPR17 and the Chemokine Receptors 2 and 4: New Evidence

**DOI:** 10.3390/ijms24010261

**Published:** 2022-12-23

**Authors:** Simona Daniele, Simona Saporiti, Stefano Capaldi, Deborah Pietrobono, Lara Russo, Uliano Guerrini, Tommaso Laurenzi, Elham Ataie Kachoie, Luca Palazzolo, Vincenzo Russo, Maria Pia Abbracchio, Ivano Eberini, Maria Letizia Trincavelli

**Affiliations:** 1 Dipartimento di Farmacia, Università di Pisa, Via Bonanno 6, 56126 Pisa, Italy; 2 Dipartimento di Scienze Farmacologiche e Biomolecolari, Università degli Studi di Milano, Via Balzaretti 9, 20133 Milan, Italy; 3 Dipartimento di Biotecnologie, Università degli Studi di Verona, Strada Le Grazie 15, 37134 Verona, Italy; 4 Cancer Gene Therapy Unit, Program of Immunology and Bio Immuno Gene Therapy of Cancer, Division of Molecular Oncology Scientific, Institute San Raffaele, 20132 Milan, Italy; 5 Laboratorio di Farmacologia Molecolare e Cellulare Della Trasmissione Purinergica, Dipartimento di Scienze Farmaceutiche, Università Degli Studi di Milano, Via Balzaretti 9, 20133 Milan, Italy; 6 Dipartimento di Scienze Farmacologiche e Biomolecolari & Data Science Research Center (DSRC), Università degli Studi di Milano, Via Balzaretti 9, 20133 Milan, Italy

**Keywords:** chemokine, homology modeling, G protein-coupled receptor (GPCR), GPR17, molecular dynamics, protein-protein interaction

## Abstract

GPR17, a G protein-coupled receptor, is a pivotal regulator of myelination. Its endogenous ligands trigger receptor desensitization and downregulation allowing oligodendrocyte terminal maturation. In addition to its endogenous agonists, GPR17 could be promiscuously activated by pro-inflammatory oxysterols and chemokines released at demyelinating lesions. Herein, the chemokine receptors CXCR2 and CXCR4 were selected to perform both in silico modelling and in vitro experiments to establish their structural and functional interactions with GPR17. The relative propensity of GPR17 and CXCR2 or CXCR4 to form homo- and hetero-dimers was assessed by homology modelling and molecular dynamics (MD) simulations, and co-immunoprecipitation and immunoenzymatic assay. The interaction between chemokine receptors and GPR17 was investigated by determining receptor-mediated modulation of intracellular cyclic adenosine monophosphate (cAMP). Our data show the GPR17 association with CXCR2 or CXCR4 and the negative regulation of these interactions by CXCR agonists or antagonists. Moreover, GPR17 and CXCR2 heterodimers can functionally influence each other. In contrast, CXCR4 can influence GPR17 functionality, but not *vice versa*. According to MD simulations, all the dimers reached conformational stability and negative formation energy, confirming the experimental observations. The cross-talk between these receptors could play a role in the development of the neuroinflammatory milieu associated with demyelinating events.

## 1. Introduction

GPR17 is a G protein-coupled receptor (GPCR) closely related to both the purine P2Y subfamily and the cysteinyl leukotriene (CysLTs) receptors (CysLT1 and CysLT2) [1,2]. During the last 15 years, some controversial studies about the GPR17 pharmacological profile have been published creating several discussions among the scientific community [3,4]. Nevertheless, there is evidence that GPR17 is activated by the uracil-nucleotides UDP, UDP-glucose and UDP-galactose and by cysteinyl-leukotrienes (LTC_4_, LTD_4_, and LTE_4_) [1,4,5,6]. These GPR17 endogenous ligands are released from damaged cells at sites of inflammation, where the receptor is upregulated, demonstrating its key role in neuroinflammatory conditions [7].

It has been largely proven that GPR17 is expressed by neural precursor cells of the oligodendrocyte lineage (NG2 glia). In particular, its expression reaches a peak when cells are in the preoligodendrocyte phase and, then, GPR17 is gradually silenced in mature myelinating oligodendrocytes [1,4,8]. Accordingly, abnormal GPR17 overexpression in late oligodendrocyte progenitor cells (OPCs) in vitro [9] and in transgenic mice results in the loss of oligodendrocytes as well as in myelination arrest [10]. As a result of these findings, GPR17 is now widely considered as a new useful marker to label NG2 progenitor cells up to the preoligodendrocyte stage and it is recognized as a putative regulator of myelination [4,11,12,13,14].

Therefore, GPR17 needs to be downregulated to permit oligodendrocyte terminal maturation [4,15] and this event, that is mediated by the interaction with its agonists, leads to the rapid receptor phosphorylation and to the initiation of the desensitization process typical of GPCRs [4,16,17]. Nevertheless, slight differences in the desensitization kinetics and intracellular pathways activation have been observed. These differences depend on the receptor agonists and demonstrate that the GPR17 functions strictly depend on extracellular stimuli. Accordingly, the biased agonism is a key aspect that must be deeply investigated [4,17].

In addition to its endogenous classic agonists, GPR17 also responds to emergency signals (e.g., oxysterols), as well as to other related receptors involved in inflammatory responses, such as the chemokine receptor CXCR2 [18]. Recently, it has been demonstrated that the stromal-derived factor 1 (SDF-1), the endogenous ligand for CXCR4 [19,20,21,22,23] and CXCR7, can transactivate GPR17 in vitro with nanomolar affinity [24]. Thus, GPR17 promiscuously responds to different signaling molecules, depending on specific pathophysiological conditions and emergency situations [4]. These recent findings highlight the heterogeneity and complexity of GPR17 pharmacology and changes the classical paradigm on the “monogamous” interaction between a GPCR and a specific class of natural ligands [4,25].

Moreover, GPR17 functional responses might depend on its heterodimerization with other receptors, including P2Y and CysLT receptors [4,26]. Indeed, receptor dimerization has been widely demonstrated to play a key role in the pharmacological properties of GPCRs [27]. As reported for a continuously increasing number of GPCRs [28], several chemokine receptors operate as homo- or hetero-dimers or even oligomers of higher order [29,30,31]. Oligomerization leads to functional changes in the cooperativity of ligand binding mechanisms, intracellular transport of GPCRs, or activation of different signaling pathways [32,33,34,35]. For example, chemokine receptors form heterodimers in complex with galectin ones, playing a role in chronic inflammation [36]. Recent findings showed also that CXCR7 and CXCR4 are able to heterodimerize recruiting intracellular proteins involved in GPCRs desensitization. This process finally results in the transcription of inflammatory factors and oncogenes [37]. Furthermore, experimentally solved structures of homodimeric CXCR4 in complex with different antagonists are currently available in the Protein Data Bank [38], as a further evidence of the class A GPCRs ability to homodimerize.

Based on the evidence that GPR17 could be promiscuously activated by pro-inflammatory oxysterols and chemokines [24] released at demyelinating lesions, in this work, we investigated the ability of chemokine receptors CXCR2 and CXCR4 to form oligomers with GPR17, via both in silico approaches and in vitro pilot experiments. The final aim of this work is to establish the potential structural and functional interactions among CXCR2 or CXCR4 and GPR17.

## 2. Results

### 2.1. Physical Interaction between GPR17 and CXCR2 Receptor

**Figure 1:**
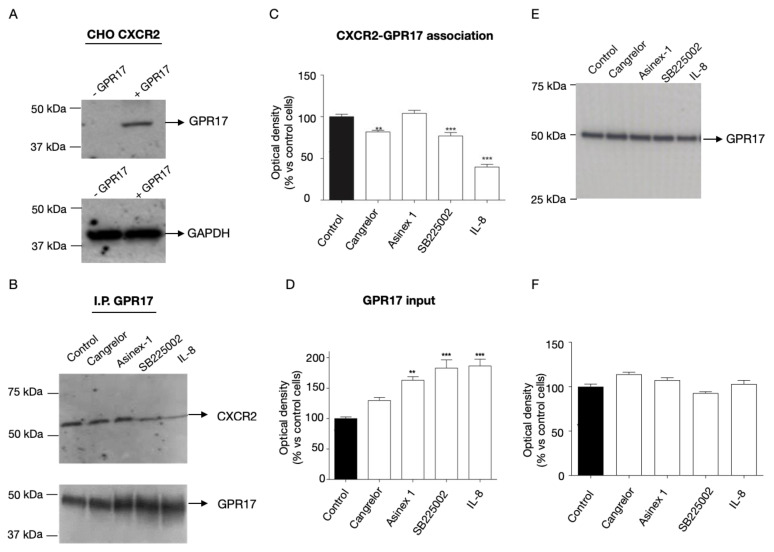
Interaction of CXCR2 and GPR17 by western blot/co-immunoprecipitation experiments. (**A**) Lysates from CHO cells stable transfected with CXCR2 and transiently transfected with GPR17 were analyzed using an anti-GPR17 antibody. GAPDH was the loading control. (**B**–**D**) CHO cells, co-expressing CXCR2/GPR17 receptors, were incubated with Cangrelor, Asinex 1, SB2250002, or IL-8 for 30 min. Lysates were immunoprecipitated with an anti-GPR17 antibody and then subjected to western blot analysis using a specific antibody for CXCR2 (**B**,**C**) or GPR17 (**B**,**D**). Representative western blots (**B**), densitometric analyses of the GPR17::CXCR2 heterodimers (**C**) and immunoprecipitated GPR17 (input signals, **D**) are shown. (**E**,**F**) CHO cells, co-expressing CXCR2/GPR17 receptors, were incubated with Cangrelor, Asinex 1, SB2250002, or IL-8 for 30 min. Cell lysates were subjected to western blot analysis using a specific antibody for GPR17. Immunoreactive bands were obtained by ImageJ program. The densitometric analysis values gave mean arbitrary units that were set to 100% for untreated control cells, and the data relative to treated cells were expressed as percentage of the mean value of optical density of control cells. The significance of the differences was determined with a one-way ANOVA with Bonferroni post-test: ** *p* < 0.01, *** *p* < 0.001 vs. control.

To verify the physical interaction between GPR17 and CXCR2, a co-immunoprecipitation and ELISA assays were performed. To this aim, we used a Chinese hamster ovary (CHO) cell line stably expressing CXCR2. This was previously validated by our research group [39] and was here transiently transfected with GPR17 (Figure 1A). The physical interaction between GPR17 and CXCR2 was then evaluated by immunoprecipitation using anti-GPR17 antibody and blotting using an anti-CXCR2 antibody, in response to a 30 min receptor activation by selective agonists and antagonists: Cangrelor (GPR17 receptor antagonist, 1 nM), Asinex 1 (GPR17 receptor agonist, 5 nM), SB225002 (CXCR2 receptor antagonist, 1 μM) and IL-8 (CXCR2 receptor agonist, 10 nM).

The detected CXCR2 bands appeared to be modulated by the pharmacological treatment (Figure 1B,C), while no immunoreactive bands specific for CXCR2 were evidenced in GPR17-immunoprecipitates from cells only expressing GPR17 (Supplementary Figure S1A). These data suggest that the indicated treatments do affect CXCR2 interaction with GPR17. Noteworthy, in GPR17 immunoprecipitates (Figure 1D), the immunoreactive bands appeared to be modulated upon cell treatment with the Asinex 1, IL-8 and SB225002 (input signals, Figure 1D) but this effect is not ascribable to changes in receptor expression (Figure 1E,F). These data suggest that GPR17 antibody can better recognize the receptor when the co-expressing cells are treated with one of the three compounds.

**Figure 2:**
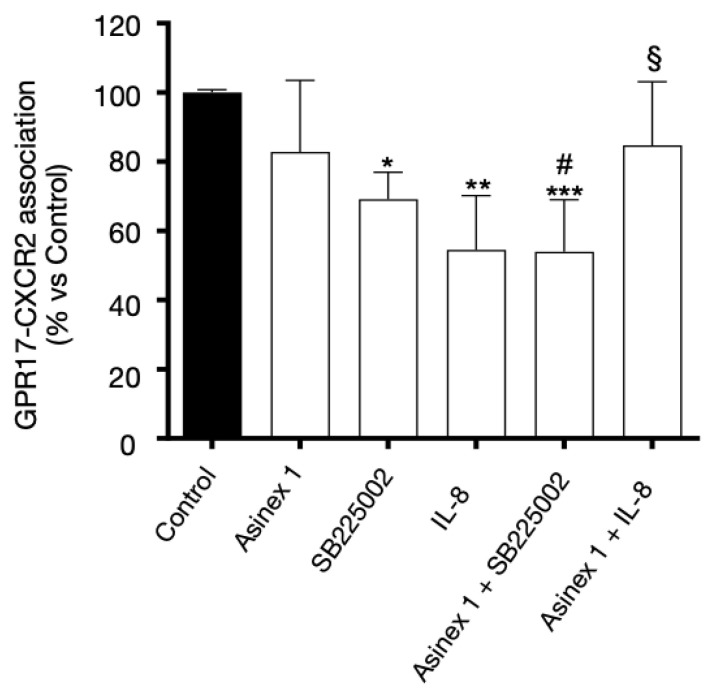
Interaction of CXCR2 and GPR17 by immunoenzymatic assay. CHO cells, stably transfected with CXCR2, were transiently transfected with GPR17, and treated with the indicated CXCR2 and GPR17 agonists or antagonists for 30 min. The levels of the GPR17::CXCR2 complex were quantified on these cell lysates (30 μg) using an antibody specific for CXCR2 by immunoenzymatic assay, as reported in the Methods section. Blanks were obtained in the absence of the primary antibody. The data are expressed as percentage of control set to 100% (mean ± SEM, N = 3). The significance of the differences was determined with a one-way ANOVA with Bonferroni post-test: * *p* < 0.05, ** *p* < 0.01, *** *p* < 0.001 vs. control; # *p* < 0.05 vs. SB225002 alone; § *p* < 0.05 vs. IL-8 alone.

To further investigate the GPR17::CXCR2 interaction, an immunoenzymatic assay on GPR17 expressing CHO-CXCR2 cells was performed (Figure 2). The results confirmed that the receptors’ association was reduced in the presence of CXCR2 ligands, but not affected by GPR17 modulation. When the two receptors were stimulated simultaneously (i.e., IL-8 + Asinex 1), the inhibitory effect of IL-8 on heterodimers formation was almost completely lost. In contrast, when the GPR17 agonist was combined with the CXCR2 antagonist SB225002, the significant decrease in GPR17::CXCR2 interaction persisted. Overall, these results suggest that GPR17 agonist can modulate differently the degree of GPR17::CXCR2 interaction in response to CXCR2 agonist vs. antagonist. Of note, no specific signal relative to CXCR2 antibody was detected on anti-GPR17-precoated wells using lysates of only GPR17-expressing cells (Supplementary Figure S1B).

### 2.2. Physical Interaction between GPR17 and CXCR4 Receptor

**Figure 3:**
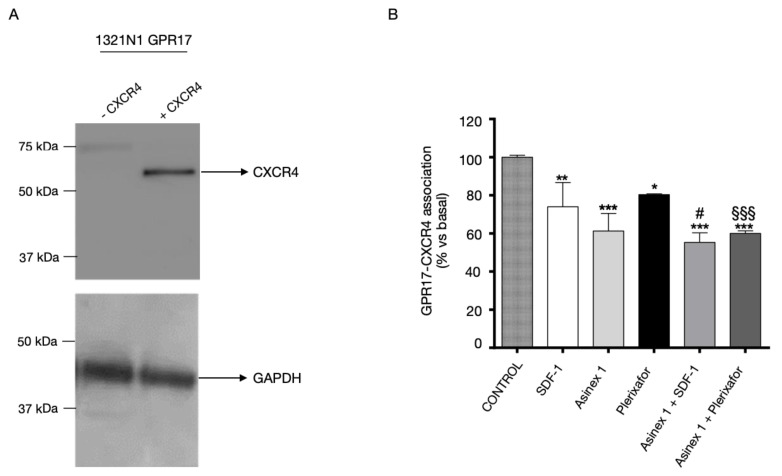
Interaction between CXCR4 and GPR17. (**A**) Western blot on cell lysates of 1321N1 stably transfected with GPR17 and transiently transfected with human CXCR4. GAPDH was the loading control. (**B**) 1321N1 cells, stably expressing GPR17, were transiently transfected with CXCR4, and treated with the indicated agonists and antagonists for 30 min. Quantification of GPR17::CXCR4 levels in these cell lysates (30 μg) was performed using an antibody specific for CXCR4 by a specific immunoenzymatic assay, as reported in the Methods section. Blanks were obtained in the absence of the primary antibody. The data are expressed as percentage of control set to 100% (mean ± SEM, N = 3). The significance of the differences was determined with a one-way ANOVA with Bonferroni post-test: * *p* < 0.05, ** *p* < 0.01, *** *p* < 0.001 vs. control; # *p* < 0.05 vs. SDF-1 alone; §§§ *p* < 0.001 vs. Plerixafor alone.

To verify the physical interaction between GPR17 and CXCR4, the 1321N1 cells, stably expressing HA-tag GPR17 [16,17], were transiently transfected with CXCR4. The efficiency of the transfection was evaluated by western blot experiment, as shown in Figure 3A. The CXCR4::GPR17 heterodimers formation after 30 min with SDF-1 (CXCR4 and GPR17 agonist, 100 ng/mL), Asinex 1 (GPR17 agonist, 5 nM) and Plerixafor (CXCR4 antagonist, 500 nM) was assessed by an enzyme-linked immunosorbent assay (ELISA).

As shown in Figure 3B, the receptors’ association was reduced by both GPR17 selective and promiscuous CXCR4 and GPR17 agonist SDF-1. The inhibitory effect was maintained when Asinex 1 and SDF-1 were administered together. Moreover, the combination of the GPR17 agonist, Asinex 1, with Plerixafor did not change the significant decrease in GPR17::CXCR4 interaction (Figure 3). Overall, these data suggest that the modulation of GPR17 and/or CXCR4, separately or simultaneously, affects the heterodimers content.

Of note, no specific signal was detected on anti-GPR17-precoated wells using lysates of only GPR17-expressing cells (Supplementary Figure S2), thus confirming the specificity of GPR17::CXCR4 interaction in the immunoenzymatic assay. Unfortunately, the same CXCR4 antibody was not suitable to be employed in co-IP experiments to confirm the receptors’ interaction.

### 2.3. Functional Interaction between GPR17 and CXCR2 Receptor

GPR17 and CXCR2 act similarly to classic GPCRs, with a specific pharmacological profile determined by highly specific ligands. Upon ligand binding to the receptor, GPR17 ligands induce a time- and concentration-dependent homologous desensitization (i.e., receptor decrease of functionality upon treatment with the same receptor ligand) [16]. In a similar way, CXCR2 shows the typical desensitization kinetics of GPCRs [40], characterized by rapid receptor phosphorylation, loss of response and downregulation [41]. As demonstrated for several GPCRs, the functional activity of CXCRs may also be regulated through heterologous desensitization, i.e., by the loss of response trans-induced by ligands of a different receptor co-expressed in the cells. In this process, if the first GPCR is activated, initiating a signaling pathway, the inactivation of an unrelated GPCR is promoted in the absence of its ligand [42]. In this sense, the ability of CXCR2 to modify GPR17 functionality, and *vice versa*, was examined by determining the receptor-mediated modulation of intracellular cyclic adenosine monophosphate (cAMP) levels upon prolonged receptor stimulation with the respective ligands. The regulation of GPCR functional activity, including cross-regulation between different GPCRs, may also take place at different levels, such as receptor trafficking in membrane, expression or positioning of scaffold proteins (GRKs, arrestins), and the control of receptor expression (in this respect, see Discussion section). Nevertheless, the assessment of cAMP production was an interesting starting point considering the pivotal role of this second messenger in directing cellular processes mediated by these GPCRs.

**Figure 4:**
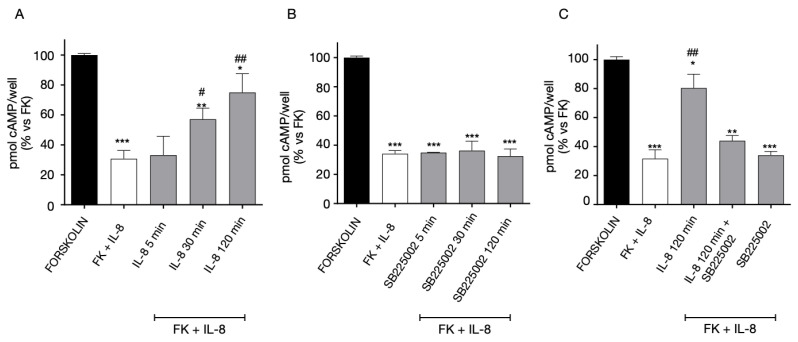
Homologous desensitization of CXCR2. CHO cells, stably transfected with CXCR2, were treated with FK in the absence (black bar) or in the presence of IL-8 (10 nM) for 15 min (white bar). Aliquots of cells (grey bars) were pre-treated with IL-8 10 nM (**A**) or SB225002 1 μM (**B**) for different times (5–120 min), washed, and then stimulated with FK in the presence of IL-8 (10 nM) for 15 min. (**C**) CHO stably expressing CXCR2, were pre-treated for 120 min with IL-8 (10 nM) or SB225002 (1 μM), alone or in combination, washed, and then stimulated with FK in the presence of IL-8 (10 nM). cAMP quantification was performed via a radioligand binding assay. The data are expressed as percentage of pmol cAMP/well versus FK. The significance of the differences was determined with a one-way ANOVA with Bonferroni post-test: * *p* < 0.05, ** *p* < 0.01, *** *p* < 0.001 vs. FK; # *p* < 0.05, ## *p* < 0.01 vs. FK+IL-8.

As a first step, homologous CXCR2 desensitization was evaluated quantifying receptor functional response to the agonist, after a pre-treatment of CXCR2-transfected cells for different times with the agonist IL-8 (Figure 4A), or the antagonist SB225002 (Figure 4B). As shown in Figure 4A, CXCR2 presents the typical desensitization kinetics of GPCRs upon pre-treatment with their agonist, IL-8, starting from 30 min of cell pre-incubation.

In Figure 4B, the cells were pre-treated for different times (5-30-120 min) with SB225002, then washed and stimulated with FK+IL-8 for 10 min. The results showed that CXCR2 functionality is not affected by treating cells with its selective antagonist, SB225002 (Figure 4B). Then, in further experiments (Figure 4C) the cells were first pre-treated for 120 min with IL-8, in the presence of the selective CXCR2 antagonist, SB225002, washed and stimulated again with IL-8 in the presence of forskolin (FK). The IL-8-induced desensitization of CXCR2 was almost completely prevented by cells pre-incubation with the receptor antagonist SB225002 (Figure 4C). These data evidence that the time-dependent decrease in FK-inhibition is mediated by CXCR2.

**Figure 5:**
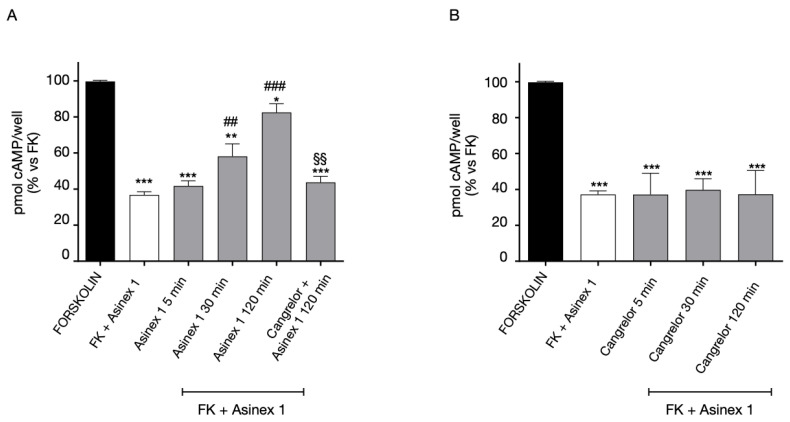
Homologous desensitization of GPR17. (**A**) 1321N1 cells, stably expressing WT GPR17, were treated with FK in the absence (black bar) or in the presence of Asinex1 (5 nM) for 15 min (white bar). Aliquots of cells (grey bars) were pre-treated for different times (5–120 min) with Asinex 1, or Asinex 1 and Cangrelor 1 nM simultaneously, and then stimulated with FK in the presence of Asinex 1 (5 nM) for 15 min. (**B**) 1321N1 cells, stably expressing WT GPR17, were pre-treated for different times (5–120 min) with Cangrelor 1 nM, and then stimulated with FK in the presence of Asinex 1 (5 nM) for 15 min (grey bars). cAMP quantification via a radioligand assay was performed in lysates of these cells. The data are expressed as percentage of pmol cAMP/well versus FK. The significance of the differences was determined with a one-way ANOVA with Bonferroni post-test: * *p* < 0.05, ** *p* < 0.01, *** *p* < 0.001 vs. FK; ## *p* < 0.01, ### *p* < 0.001 vs. FK + Asinex 1; §§ *p* < 0.01 vs. 120 min Asinex 1-induced desensitization.

For what concerns GPR17 homologous desensitization, the cAMP experiments confirmed our previous results [16]. In particular, the data evidenced that the receptor agonist Asinex 1 induced a time-dependent loss of GPR17 functionality (Figure 5A). The Asinex 1-induced desensitization of GPR17 was significantly inhibited by the receptor antagonist Cangrelor (Figure 5A) demonstrating that that the time-dependent decrease of FK-inhibition is mediated by GPR17.

In contrast, no significant changes in receptor-mediated responses were evidenced when cells were challenged with the GPR17 antagonist Cangrelor (Figure 5B).

**Figure 6:**
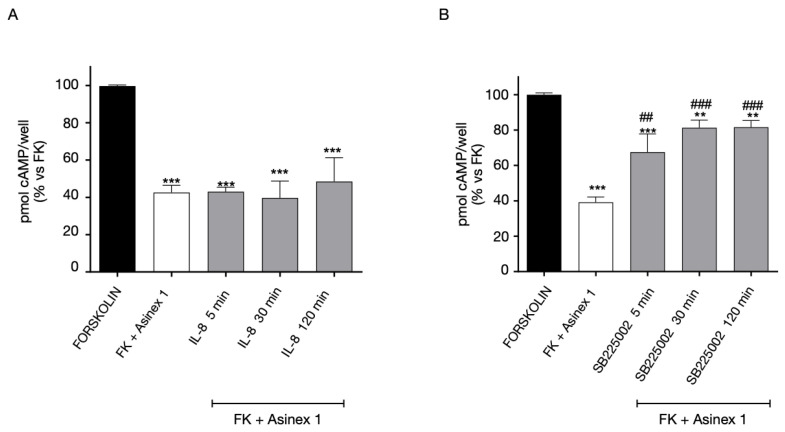
Heterologous desensitization of GPR17 receptor. CHO cells, stably expressing CXCR2, were transiently transfected with GPR17, and treated with FK in the absence (black bar) or in the presence of Asinex1 (5 nM) for 15 min (white bar). Aliquots of cells (grey bars) were pre-treated for different times (5–120 min) with IL-8 10 nM (**A**) or SB225002 1 μM (**B**), washed, and then stimulated with FK in the presence of Asinex 1 (5 nM) for 15 min. cAMP quantification was assessed via a radioligand assay in lysates of these cells. The data are expressed as percentage of pmol cAMP/well versus FK. The significance of the differences was determined with a one-way ANOVA with Bonferroni post-test: ** *p* < 0.01, *** *p* < 0.001 vs. FK; ## *p* < 0.01, ### *p* < 0.001 vs. FK + Asinex 1.

Then, we evaluated heterologous GPR17 and CXCR2 cross-regulation in cells co-expressing both the receptor subtypes. To investigate the regulation of GPR17 functionality in response to CXCR2 modulation, cells were pre-treated for different times with CXCR2 ligands, and after being washed, stimulated with the GPR17 agonist, Asinex 1, in order to assess potential changes in GPR17 responsiveness (Figure 6). GPR17 functional response is maintained after cells stimulation with the CXCR2 receptor agonist, IL-8, (Figure 6A). In contrast, a pre-stimulation with the CXCR2 antagonist, SB225002, caused a significant decrease in GPR17 responses, indicating a GPR17 desensitization (Figure 6B). Of note, Asinex 1 had no effects on CHO cells expressing CXCR2 only (Supplementary Figure S3A), and IL-8 did not show significant effects on GPR17 functionality in 1321N1 cells-expressing GPR17 only (Supplementary Figure S3B). These results demonstrate that, whereas no significant effects on GPR17 functional response is observed in the presence of CXCR2 agonist, the functional response of GPR17 is impaired when CXCR2 is bound by its antagonist in cells co-expressing CXCR2-GPR17.

**Figure 7:**
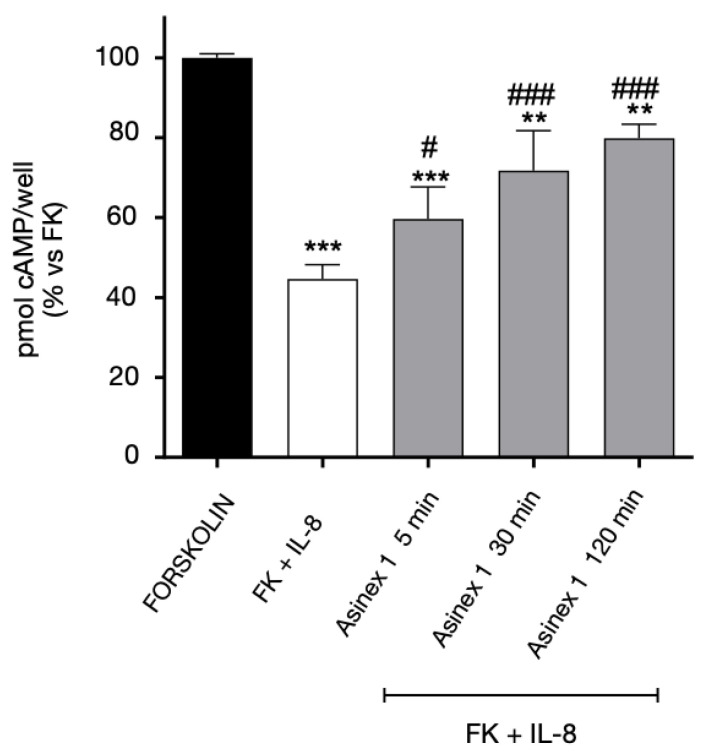
Heterologous desensitization of CXCR2 receptor. CHO cells, stably expressing CXCR2, were transiently transfected with WT GPR17, and treated with FK in the absence (black bar) or in the presence of IL-8 (10 nM) for 15 min (white bar). Aliquots of cells (grey bars) were pre-treated for different times (5–120 min) with Asinex 1 (5 nM), washed, and then stimulated with FK in the presence of IL-8 (10 nM) for 15 min. cAMP quantification was assessed via a radioligand assay in lysates of these cells. The data are expressed as percentage of pmol cAMP/well versus FK. The significance of the differences was determined with a one-way ANOVA with Bonferroni post-test: ** *p* < 0.01, *** *p* < 0.001 vs. FK; # *p* < 0.05, ### *p* < 0.001 vs. FK+IL-8.

In addition, we investigated if CXCR2 functional responsiveness could be modified by GPR17 activation. When GPR17-CXCR2 co-transfected cells were pre-treated with the GPR17 agonist Asinex 1, responses of CXCR2 significantly decreased, indicating a desensitization phenomenon of this receptor (Figure 7). These data suggest that CXCR2 functionality is modulated when GPR17 is stimulated by its agonist.

### 2.4. Functional Interaction between GPR17 and CXCR4 Receptor

**Figure 8:**
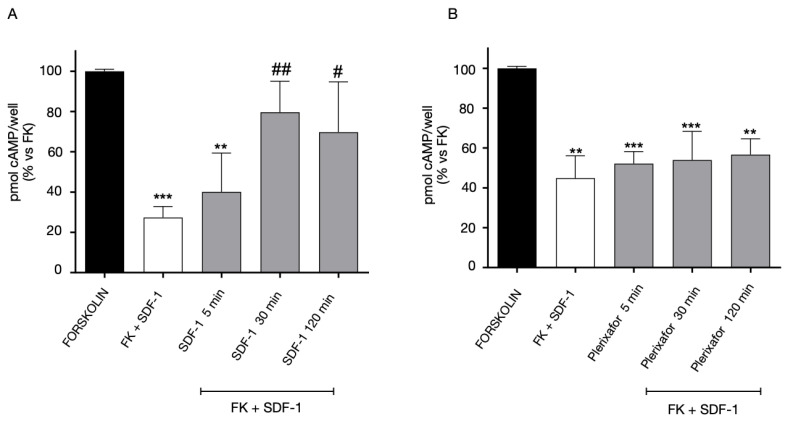
Homologous desensitization of CXCR4. 1321N1 cells, transiently transfected with CXCR4, were treated with FK in the absence (black bar) or in the presence of SDF-1 (100 ng/mL) for 15 min (white bar). Aliquots of cells (grey bars) were pre-treated for different times (5–120 min) with SDF-1 100 ng/mL (**A**) or Plerixafor 500 nM (**B**), washed, and then stimulated with FK in the presence of SDF-1 (100 ng/mL) for 15 min. cAMP quantification was assessed via a radioligand binding assay in lysates of these cells. The data are expressed as percentage of pmol cAMP/well versus FK. The significance of the differences was determined with a one-way ANOVA with Bonferroni post-test: ** *p* < 0.01, *** *p* < 0.001 vs. FK; # *p* < 0.05, ## *p* < 0.01 vs. FK+SDF-1.

The functional interaction between CXCR4::GPR17 was evaluated by assessing the amount of intracellular cAMP in response to GPR17 or CXCR4 agonists. First, the homologous desensitization experiments were performed in 1321N1 cells expressing only CXCR4 (Figure 8). The cells were treated for different times (5–120 min) with SDF-1 (Figure 8A) or Plerixafor (Figure 8B), washed, and then stimulated with FK in the absence or presence of its agonist SDF-1. As shown in Figure 8A, upon pre-treatment with SDF-1, CXCR4 showed the typical desensitization kinetics of GPCRs. In particular, the agonist was able to significantly decrease the CXCR4 functionality starting from 30 min of pre-incubation. In contrast, CXCR4 functionality was not affected by the pre-treatment with the selective antagonist, Plerixafor (Figure 8B).

**Figure 9:**
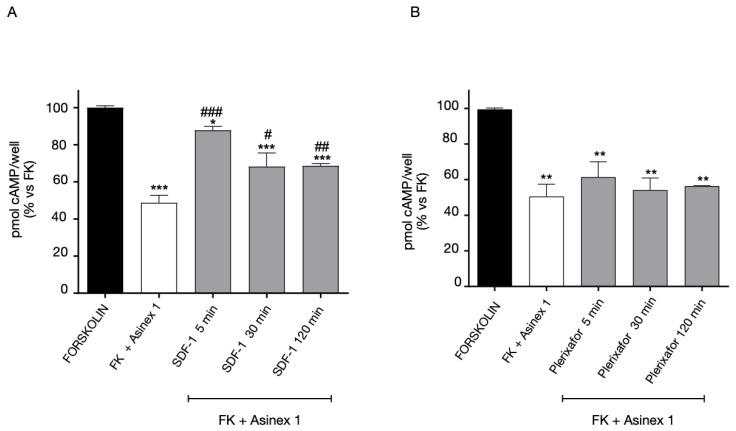
Heterologous desensitization of GPR17 receptor. 1321N1 cells, stably expressing GPR17, were transiently transfected with CXCR4, and treated with FK in the absence (black bar) or in the presence of Asinex 1 (5 nM) for 15 min (white bar). Aliquots of cells (grey bars) were pre-treated for different times (5–120 min) with SDF-1 100 ng/mL (**A**) or Plerixafor 500 nM (**B**), washed, and then stimulated with FK in presence of Asinex 1 (5 nM) for 15 min. Quantification was assessed via a radioligand assay in lysates of these cells. The data are expressed as percentage of pmol cAMP/well versus FK. The significance of the differences was determined with a one-way ANOVA with Bonferroni post-test: * *p* < 0.05, ** *p* < 0.01, *** *p* < 0.001 vs. FK; # *p* < 0.05, ## *p* < 0.01, ### *p* < 0.001 vs. FK + Asinex 1.

Then, the experiment was set up in cells expressing both GPR17 and CXCR4. The 1321N1 cells co-expressing the receptors were pre-treated for different times (5-120 min) with CXCR4 ligands, and then, stimulated with Asinex 1 (Figure 9). The pre-treatment with the agonist SDF-1 (Figure 9A) induced a rapid GPR17 desensitization. In contrast, when the cells were pre-challenged with the CXCR4 antagonist, Plerixafor, GPR17 remains functionally active, (Figure 9B).

**Figure 10:**
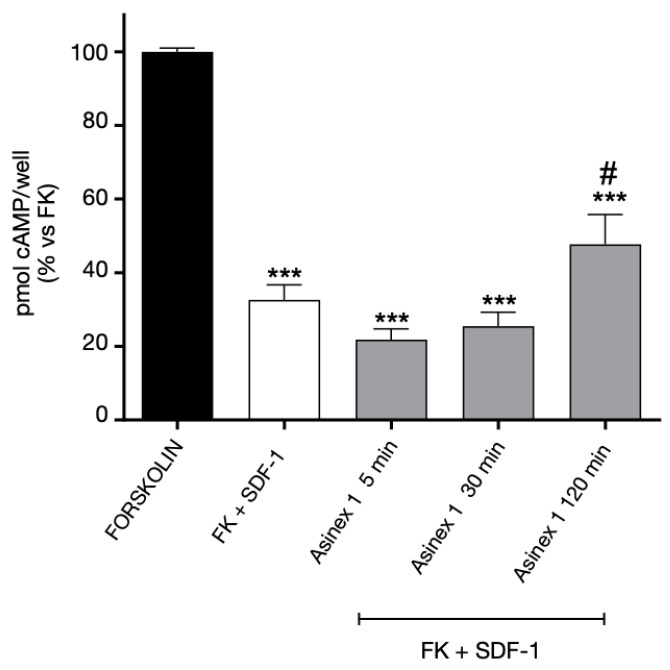
GPR17/CXCR4 mixed responses. 1321N1 cells, stably expressing GPR17, were transiently transfected with CXCR4, and treated with FK in the absence (black bar) or in the presence of SDF-1100 ng/mL) for 15 min (white bar). Aliquots of cells (grey bars) were pre-treated for different times (5–120 min) with Asinex 1 5 nM, washed, and then stimulated with FK in the presence of SDF-1 (100 ng/mL) for 15 min. cAMP quantification was assessed via a radioligand assay in lysates of these cells. The data are expressed as percentage of pmol cAMP/well versus FK. The significance of the differences was determined with a one-way ANOVA with Bonferroni post-test: *** *p* < 0.001 vs. FK; # *p* < 0.05 vs. FK+SDF-1.

Finally, 1321N1 CXCR4-GPR17 co-transfected cells were pre-treated with the GPR17 agonist, Asinex 1 (Figure 10A), and then stimulated with SDF-1 (a promiscuous CXCR4 and GPR17 agonist). The results showed that the SDF-1-evoked responses (mediated by the activation of both GPR17 and CXCR4) were maintained and slightly affected after 120 min of pre-treatment with Asinex 1 (Figure 10). Of note, Asinex 1 did not significantly affect the functionality of CXCR4 on 1321N1 cells expressing CXCR4 only (Supplementary Figure S3, panel C), thus denoting the specificity of its effects only in cells that co-express GPR17 and CXCR4. Nevertheless, the interpretation of Figure 9 is difficult due to the lack of an effective control because of the promiscuity of SDF-1.

Overall, our results suggest that the prolonged stimulation of CXCR4 can modulate GPR17 responses. In contrast, upon prolonged CXCR4 blockage, GPR17 functional responses are almost completely maintained. Finally, upon prolonged GPR17 stimulation, CXCR4 functionality is maintained.

### 2.5. In Silico Homology Modeling and Molecular Dynamics Simulations

In order to evaluate the physical (thermodynamic) stability of CXCR4, CXCR2 and GPR17 in their homo- and hetero-dimeric forms and to further investigate their structural behavior, the three-dimensional (3D) structure of six dimers and three monomers was produced as described. Specifically, nine systems were generated, including: CXCR4, CXCR2 and GPR17 in a monomeric form; CXCR4::CXCR4, CXCR2::CXCR2 and GPR17::GPR17 homodimers; CXCR4::CXCR2, CXCR2::GPR17 and CXCR4::GPR17 heterodimers (Supplementary Figure S4). Monomeric receptors were simulated with the only purpose of dimer formation energy analysis (please, see the next paragraph). In Supplementary Figure S5, a structural superposition between the X-ray structure of CXCR4 homodimer and all the other complexes is reported with the aim to compare the dimeric models with an experimentally solved structure. Accordingly, an overall RMSD of 3.52 Å and 2.85 Å was obtained for the homodimers and heterodimers superposition, respectively. The main differences are related to loops variability and some shift of the helices, but globally the orientation of dimers is comparable.

As further described in the “Materials and Methods” section, the 3D structure of all these GPCRs has been experimentally solved, except for GPR17. In this case, a homology modeling procedure was applied to predict its structure. However, only very recently, a Cryo-EM structure of this receptor has been published (PDB ID: 7Y89) [43]. So, to assess the accuracy of our model, we performed a structural analysis of our and the experimentally solved GPR17 structures (Supplementary Figure S6). Accordingly, after superposition, the two structures present a RMSD value of 2.85 Å, with highly conserved transmembrane regions. Some differences have been observed for extracellular loops and the N-terminal portion of TM5, that in our model is partially unfolded with respect to the Cryo-EM structure, with a local RMSD of 3.27 Å. Considering the whole TM5 helix instead, the RMSD value is 1.85 Å. Furthermore, a GPR17 homodimer was generated superposing the Cryo-EM structure to our dimeric complex and, after energy minimization to a 0.1 kcal/mol/Å^2^ RMS gradient, the minimized complex was superposed to the starting model showing a total RMSD of 2.5 Å and suggesting very comparable orientations (Supplementary Figure S7). Thus, this small difference in TM5 helix should not affect neither the orientation of the monomer in the dimeric complex nor the quality of MD results. The stability of all the dimeric complexes was evaluated along the 250 ns MD production phases simulated in replicate. According to this analysis (Supplementary Figure S8A), all the complexes reach a conformational stability, expressed as RMSD plateau, after about 20 ns of simulation. Monomers resulted to be stable along replicas too, confirming in another way the reliability of the simulated structures. RMSF was computed to evaluate fluctuation profiles and proteins flexibility during the dynamics. As expected, most of the fluctuation was recognized for protein loops, both intracellular and extracellular, that are known to be very flexible domains, typically unstructured (Supplementary Figure S8B). Thus, considering not particularly relevant events of unfolding or geometric instability, the modeled complexes can be considered reliable for further investigations.

### 2.6. Analysis of the Dimerization Interface

**Figure 11:**
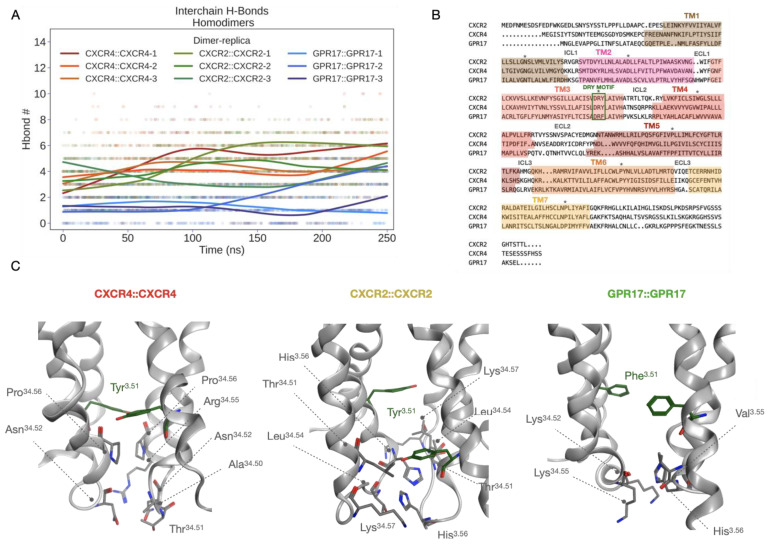
The interaction interface in homodimers and the sequence alignment between CXCR2, CXCR4 and GPR17. (**A**) Inter-chain H-bonds number by replica vs. simulation time. In addition to the scatterplot, a LOWESS nonparametric interpolation is shown (continuous lines). (**B**) Sequence alignment between CXCR2, CXCR4 and GPR17. TM regions, ICLs and ECLs are reported in the picture together with the “DRY” motif highlighted by the green square. “*” represent the conserved residue 50 in each helix according to the Ballesteros-Weinstein numbering. (**C**) The structural representation of interactions between ICL2 regions and the network of Tyr^3.51^ in “DRY” motif. On the right, in GPR17 homodimer the orientation of the corresponding Phe^3.51^ that does not participate to the dimerization. Reported structures, represented as grey ribbons, are medoids isolated from the most populated cluster computed for all replicas. Residue atoms are shown as colored sticks according to the atom color code; Tyr/Phe^3.51^ carbons are colored in green; hydrogens are not displayed for clarity.

To further evaluate the stability of complexes and to further elucidate the dimerization process, the dimerization interface was analyzed by computing hydrogen bonds (H-bonds), salt bridges, π-π and π-cation interactions. The analysis revealed that H-bonds represent the main actors in the formation of dimers, since the other types of interactions occur only at a very low extent to determine significant differences (Supplementary Figure S9). A cumulative list of H-bonds identified along the replicas with an occupancy of at least 20% is reported in Supplementary Tables S1 and S2. According to this result, a comparable set of interactions was identified between homodimers, including a very similar number of H-bonds, especially for CXCR2::CXCR2 and CXCR4::CXCR4 (Figure 11A). Moreover, in both complexes, the residue Tyr^3.51^, that is in the conserved “DRY” motif in TM3 (Asp^3.49^, Arg^3.50^, Tyr^3.51^ according to Ballesteros-Weinstein numbering) [44] was found to interact with Leu^4.41^ (CXCR2) and Pro^34.56^ (CXCR4). This motif is typical of class A GPCRs and is involved both in the stabilization of the internal structure of receptors and in the interaction with the C-terminus of G protein after receptor activation [45], thus suggesting a putative role of this region also in regulating the dimerization process. Although in GPR17 this region is mutated in “DRF” and the corresponding Phe^3.51^ is not part of the interaction interface, an implicit cross-validation of our GPR17 model is given through the results published by Fang Ye and colleagues on the Cryo-EM structure of GPR17 [43]. In fact, despite the coordinates are still missing in PDB, a similar orientation of “DRF” motif with respect to that present in our model is described. Furthermore, in all homodimers the most of contacts occurs in ICL2, even though, as shown by the sequence alignment reported in Figure 10B, the residues located in this loop are only poorly conserved among CXCR2, CXCR4 and GPR17. In Figure 10C, the structural representation of interactions occurring in the ICL2 region is reported for the three homodimers together with those bonds made by Tyr^3.51^. According to this result, the sequence variability observed in this region could influence the promiscuity of receptors and the choice of the partner for the homo- or hetero-dimerization.

**Figure 12:**
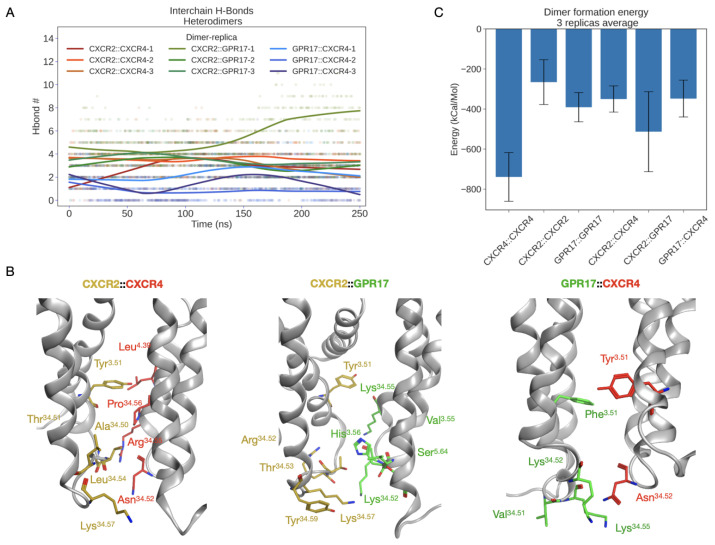
The interaction interfaces in heterodimers and the dimer formation energy. (**A**) Inter-chain H-bonds number by replica vs. simulation time. In addition to the scatterplot, a LOWESS nonparametric interpolation is shown. (**B**) The structural representation of interactions between ICL2 regions and the network of Tyr/Phe^3.51^ in “DRY” motif. On the right, in the GPR17::CXCR4 heterodimer neither Phe^3.51^ or Tyr^3.51^ were found to participate to the dimerization. Reported structures, represented as grey ribbons, are medoids isolated from the most populated cluster computed for all replicas. Residue atoms are shown as sticks, with carbons colored in gold for CXCR2, in green for GPR17 and in red for CXCR4; hydrogens are not displayed for clarity. (**C**) Average dimer formation energy histogram. Values in kcal/mol represent means of energies computed for replicas, error bars correspond to the standard error of the mean (SEM) of the three replicas.

Looking at heterodimeric interfaces, a comparable number of contacts were found for CXCR2::GPR17 and CXCR4::CXCR2 complexes, suggesting that these could be the most favored ones with respect to GPR17::CXCR4 heterodimer, in which only six H-bonds were observed (Figure 12A and Supplementary Table S2). For all the heterodimers the interactions are mainly located in the ICL2, as well as in homodimers interface, and in the case of CXCR2::GPR17 and CXCR4::CXCR2 an involvement of the conserved Tyr^3.51^ was detected too, further confirming a key role of these regions in the stabilization of the complexes. However, considering the heterodimerization of CXCR4 and GPR17, neither Tyr^3.51^ in CXCR4 or Phe^3.51^ in GPR17 make interactions with the corresponding counterpart. The structural representation of ICL2 and Tyr/Phe^3.51^ is reported in Figure 12B.

### 2.7. Dimer Formation Energy Analysis

The dimer formation energy was computed. This analysis was useful to evaluate if the homo- and hetero-dimerization of CXCR2, CXCR4 and GPR17 is a thermodynamically favored process during time and if any differences exist in terms of energy between complexes. Figure 12C reports the average dimer formation energy computed between the replicas. As well as in the MM/GBSA results, the main difference was observed between the CXCR4::CXCR4 homodimer, showing the most negative energy value (approximately −700 kcal/mol), and all the other complexes. In the case of CXCR2 and GPR17 homodimers comparable energy values were detected, confirming the similar interaction mode hypothesized according to the H-bonds analysis. By carefully looking at the investigated heterodimers, a slight difference can be observed between CXCR2::GPR17 and the others. Specifically, the lower energy value of this complex (approximately −500 kcal/mol) suggests, again, that its formation could be the most favorable among all heterodimers.

Globally, this analysis confirms not only a stabilization of complexes during the MD, but it also shows that the formation of all the simulated complexes is possible according to a thermodynamic perspective, strongly supporting our experimental results.

## 3. Discussion

We investigated the relative propensity of GPR17 and the chemokine receptors CXCR2 and CXCR4 to homo- and hetero-dimerize, using both in vitro and in silico approaches. Moreover, the ability of chemokine receptors to modify GPR17 functionality and *vice versa* was studied by assessing the receptor-mediated modulation of intracellular cAMP. There is evidence that GPR17 could be promiscuously activated by pro-inflammatory oxysterols and chemokines [24] released at demyelinating lesions. Accordingly, the detailed understanding of GPR17::CXCRs cross-talk with respect to ligand recognition, signal transduction, and oligomerization properties will allow to shed light on receptors cross talk in the development and progression of the neuroinflammatory milieu. The main results of the present study are summarized as follows: (i) GPR17 associates with CXCR2 or CXCR4; (ii) the interaction of GPR17 with CXCR2 or CXCR4 is negatively regulated by CXCRs agonists or antagonists; (iii) when forming heterodimers, GPR17 and CXCR2 can functionally influence each other; (iv) CXCR4 can influence GPR17 functionality but not *vice versa*. Based on these results, we hypothesize that a cross-talk between these receptors could play a role in the development of the neuroinflammatory milieu associated with demyelinating events. The future investigation in native systems will further shed light on the biological relevance of these results.

To verify the physical interaction between GPR17 and CXCR2 receptors, co-immunoprecipitation and immunoenzymatic assays were performed using transfected cells expressing both GPCRs. CXCR2 and GPR17 were demonstrated to interact in basal conditions and upon treatment with respective agonists/antagonists. Moreover, our data demonstrate that GPR17::CXCR2 heterodimers can be modulated regardless by CXCR2 agonists or antagonists, challenging the traditional view of antagonists as inactive ligands [46]. Consistently, high concentrations of A_2A_ adenosine receptor antagonists behave as receptor agonists, decreasing D_2_ dopamine receptor expression and functionality in the brain [46]. Furthermore, the D_2_ dopamine receptor antagonist haloperidol affects the degree of A_2A_::D_2_ receptor heterodimerization [47]. In contrast, the atypical antipsychotic clozapine has no effect on A_2A_ adenosine receptor parameters, suggesting that the modulation of GPCR heterodimers is dependent on the class of drugs [47].

When the two receptors were stimulated simultaneously, the inhibitory effect of the CXCR2 agonist on heterodimers formation was almost completely lost, suggesting that GPR17::CXCR2 heterodimers may be modulated differently if both ligands are present concomitantly. In contrast, when the GPR17 receptor agonist was combined with the CXCR2 antagonist, the inhibitory effect on heterodimers formation persisted. Thus, the binding of CXCR2 agonist or antagonist seems to modulate the receptor conformation in a different way, leading to a different interaction with GPR17 only in the presence of a ligand for the latter receptor. In this sense, several studies have reported that receptor heterodimerization leads to new binding properties [48,49,50,51,52,53,54], suggesting that heterodimerization induces an alteration in the conformation of the ligand-binding site of the involved GPCRs.CXCR2 underwent homologous desensitization upon challenge with the receptor agonist. In contrast, CXCR2 functionality was not affected by the pre-treatment with its selective antagonist, SB225002, as well as the classical model in which only the agonist-occupied receptor becomes a substrate for phosphorylation by members of the GRK family [55].

Next, the ability of CXCR2 to modify GPR17 functionality and *vice versa* was examined by determining the receptor-mediated modulation of intracellular cAMP upon prolonged receptor stimulation with the respective ligands. Herein, GPR17 functionality is modulated by the presence of CXCR2, blocked by its antagonist. This suggests that the blockage of CXCR2 induces a conformational change in CXCR2::GPR17 heterodimer that affects GPR17 functionality. Of note, the loss of GPR17 functional response might depend on the agonist efficacy and potency, as well as from the activation of different signaling pathways. Although an unusual mechanism, the literature reports other examples of antagonist-induced desensitization: for example, the antipsychotic olanzapine causes desensitization of serotonin (5-HT)(2A) receptors in a rat cortical cell line [56], while pindolol, an antagonist with intrinsic sympathomimetic activity, decreases the functionality and expression of β-adrenergic receptors [57].

On the other hand, CXCR2 response decreases, indicating receptor desensitization, when GPR17 is stimulated by its agonist. These results highlight that the two GPCRs influence each other: accordingly, other studies on the interactions between CXCR1, CXCR2 and CCR5 show that CXCR1 exhibit bi-directional heterologous desensitization [58].

Then, the interaction between GPR17 and CXCR4 was examined. The CXCR4::GPR17 heterodimer was observed under basal condition and the functional properties of this complex were investigated.

Our results demonstrate that CXCR4 can influence GPR17 functionality but not *vice versa.* Consistent with our results, evidence for asymmetrical cross-regulation of CXCR4 has been proven for α1-adrenergic receptors within the heteromeric receptor complex [59,60].

It should be pointed out that our experiments explored the effects of an acute administration of GPR17 and/or chemokine ligands: chronic agonist administration may yield results which are qualitatively or quantitatively different, as suggested by recent literature [42]. Moreover, the physical and functional interactions between the receptors were examined at a single concentration of different agonists and antagonists and this experimental choice cannot capture entirely the in vivo complexity. Finally, besides a direct physical interaction between the receptors, the heterodimerization can be due to downstream signaling events too. In fact, the regulation of GPCR functional activity, including cross-regulation between different GPCRs, may take place at different levels, such as receptor trafficking in membrane, expression or positioning of scaffold proteins, and the control of receptor expression [42]. In this sense, the investigation of receptor functionality is of pivotal importance, considering the role of these receptors in demyelinating lesions, as well as our previous studies investigating GPR17 desensitization and signals in both transfected [16,17] and naïve cells [15].

In parallel to experimental studies, MD simulations were performed in replicates to evaluate in silico the stability of homo- and hetero-dimers formed by CXCR2, CXCR4 and GPR17 that were modeled upon a published dimeric structure of CXCR4 [38]. This structure was chosen for its physiological relevance of the identified dimerization interface, as demonstrated via computational methods by Rodríguez et al. [61] and experimentally by Ward and colleagues in a recently published work [62] in which, after multiple mutations in the interface, only monomeric forms of the receptor could be observed. Moreover, the Cryo-EM structure of GPR17 has been published only recently (PDB ID: 7Y89) [43]. The comparison of our GPR17 model with the cryo-EM structure showed a very high structural similarity, suggesting a high accuracy of the model that was submitted to MD. As a result, all the simulated complexes (CXCR4::CXCR4, CXCR2::CXCR2 and GPR17::GPR17 homodimers; CXCR4::CXCR2, CXCR2::GPR17 and CXCR4::GPR17 heterodimers) reach a conformational stability within the simulated time. The stability was evaluated according to geometric analysis (RMSD and RMSF) and to the analysis of interaction interfaces. A conserved interaction region was identified among the complexes, namely the ICL2, suggesting that there could be a shared homo- hetero-dimerization mechanism mediated by this loop. However, according to the sequence alignment of the receptors, a certain variability is present in ICL2. This mainly suggests that the interaction region is probably conserved because of the physical proximity of receptors in membrane, but the different aminoacidic composition, together with ligand binding, can be responsible for the preference of such partners with respect to others. Moreover, another relevant difference in the binding mode can be observed between chemokine receptors and GPR17, due to the presence of a substitution in the conserved “DRY” motif. The conserved Tyr^3.51^ in fact was found to be part of the homodimerization interface only in chemokine receptors, while in GPR17 it is replaced by Phe^3.51^ and does not participate to the interaction between monomers. Similarly, in the heterodimers formation, the Tyr^3.51^ is involved only in the case of CXCR2::CXCR4 and CXCR2::GPR17, leading us to hypothesize that the structural complementarity between CXCR4 and GPR17 could be less than between others, as confirmed also by the lower number of interactions. Moreover, the dimer formation energy was computed to better define the thermodynamic stability of complexes. As expected, the crystalized CXCR4 homodimer is the most stable, while all the other complexes show similar energy values, even if the CXCR2::GPR17 was slightly more favored. In conclusion, our in silico models strongly support the hypothesized cross talk mechanism between chemokine receptors and GPR17. Globally, this in silico study opens new perspectives to the investigation of GPCRs dimerization process and, considering the very recent GPR17 experimental structure, future investigations will be devoted to further confirm our data and the role of those residues identified as part of the dimerization interface.

**Figure 13:**
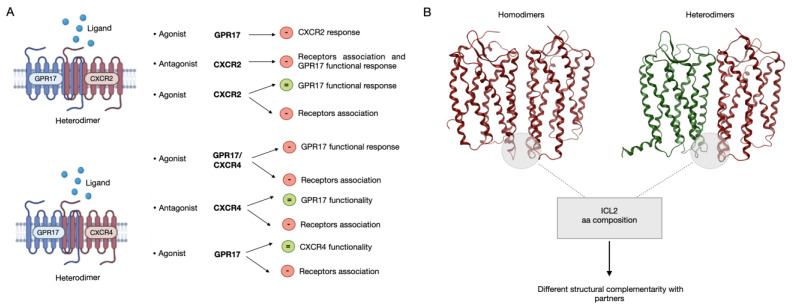
A model graphic summarizing functional heterodimerization between GPR17 and the chemokine receptors 2 and 4. The main biochemical (**A**) and in silico (**B**) results obtained in the present paper were reported. Briefly: (i) GPR17 associates with CXCR2 or CXCR4; (ii) the interaction of GPR17 with CXCR2 or CXCR4 was negatively regulated using CXCR agonists or antagonists; (iii) when forming receptor heteromers, GPR17 and CXCR2 can influence their functionality in a bi-directional way; (iv) CXCR4 can influence GPR17 functionality, but not *vice versa*. (**B**) According to in silico results, both homo- and heterodimers mainly dimerize via the ICL2 loop that presents a certain sequence variability across the receptors, thus suggesting a different structural complementarity with partners.

A model graphic summarizing our data is reported in Figure 13.

The ability of one GPCR to inhibit the activity of another GPCR can play a role in finely regulating several receptor functions, including key aspects of inflammatory cell functions, such as chemotaxis, the production of pro-and anti-inflammatory mediators and the chronicization of acute inflammatory events [42]. In this respect, future studies will clarify if differences in GPR17 heterodimerization at different stages of demyelinating diseases could play a role in the “resistance” of GPR17 to physiological desensitization, which has been shown to lead to pathological receptor upregulation and the consequent blockade of oligodendrocyte differentiation to mature myelinating cells.

It could indeed be postulated that elevated local levels of proinflammatory CXCR ligands at demyelinated sites during disease course may alter the degree of GPR17 heterodimerization and its desensitization/downregulation kinetics, leading to inability to repair lesions. These findings may thus open a new scenario in the understanding of the pathological mechanisms related to these receptors and in the identification of new pharmacological tools to restore their correct functional cross talk. Since SDF-1 accumulates at the sites of demyelination and both GPR17 and chemokine receptors are recognized molecular targets in multiple sclerosis, these results suggest, for the first time, that a cross-talk between these receptors could play a role in the development of the neuroinflammatory milieu associated with demyelination lesions.

The translation of these studies to a native cell system will surely allow for clarification regarding both the specificity of GPR17 interaction with CXCRs and its ability to cooperate with additional GPCRs, undercovering the limitations of the present results using a transfected cell model that does not completely reflect physiological conditions. A direct comparison between GPR17 expression in transfected cells and in naïve ones is challenging. Nevertheless, the GPR17 overexpression in transfected cells can mimic the receptor upregulation in demyelinating lesions or in brain damaged areas involving oligodendroglia cells [63,64,65]. The future investigation in native systems, including the degree of receptor dimerization, also in comparison with other receptor that are known to dimerize in physiological conditions, will further shed light on the biological relevance of these results. In fact, it is noteworthy that receptor hetero-dimerization, affecting surface receptor responses with un-predictable functional consequences, is an important determinant of cellular response in health and disease. Since the relative expression of different GPCRs in various cell types may differ, and consequently also the levels of receptor heterodimerization, the investigation of this issue in native systems may likely have clinical and therapeutic significance. This issue is particularly relevant for GPR17, as its role in myelination process, is strictly dependent by the fine modulation of receptor functional responses and may be crucial for the development of new therapeutic strategies in demyelinating diseases.

## 4. Materials and Methods

### 4.1. Compounds

Cangrelor (Sigma Aldrich, Milan, Italy, 1 nM) [6,8] was used as GPR17 antagonist; Asinex 1 refers to 1 (2-[[5-(2-methoxyphenyl)-4-(4-methoxyphenyl)-4H-1,2,4-triazol-3-yl]thio]-N-phenyl-propanamide; CAS 483283-39-2, previously published as ASN 02563583), was purchased from Ambinter (c/o Greenpharma, Orlèans, France) [39,66] and was used as GPR17 agonist; IL-8 (SigmaAldrich, Italy, 10 nM) [67] was used as CXCR2 agonist and SB225002 (SigmaAldrich, Italy, 1 μM) [67] was used as CXCR2 antagonist. SDF-1 (Stromal cell-derived factor 1, SigmaAldrich, Italy, 100 ng/mL) [24] was used as CXCR4 and GPR17 agonist, while plerixafor (AMD3100 octahydrochloride hydrate, SigmaAldrich, Italy, 500 nM) [68] was used as CXCR4 antagonist. FK (SigmaAldrich, Italy) was employed as adenylyl cyclase activator. Such concentrations were chosen considering the affinity of the compounds to GPR17, CXCR2 or CXCR4. For CXCR4, the unavailability of a compound that selectively targets it did not allow to deeply investigate the structural interactions of the two receptors under single CXCR4 stimulus.

### 4.2. Cell Lines and Culture

In order to test the interaction between GPR17 and CXCR2/4, and to use suitable positive and negative controls, the following cell lines were employed: (i) CHO-human stably transfected with CXCR2 [39]; (ii) 1321N1 astrocytoma stably transfected with HA-tag GPR17 [17,39]; (iii) CHO stably expressing CXCR2, and transiently transfected with GPR17; (iv) 1321N1 GPR17-expressing cells, transiently transfected with CXCR4; (v) 1321N1 GPR17-cells, transiently transfected with CXCR4; 1321N1 and CHO cells do not express any endogenous GPR17 or chemokine receptors, as evidenced by the data depicted in Figure 1A, Figure S1A and Figure 3A.

### 4.3. Plasmid Construction

For transient expression of human GPR17 (short isoform, UniProt ID: Q13304-2) and human CXCR4, the cDNA sequence of the two receptors, including the initial Met and the stop codon, were amplified by PCR with primers designed to introduce suitable restriction sites at both ends. After digestion, the amplicons were purified and ligated into the pcDNA3.1 vector. All constructs were verified by DNA sequencing.

For the transfections, the PEI method was applied. The 1321N1 GPR17-expressing cells were seeded in 24-well plates or in Petri dishes and incubated for 48 h with a solution containing 250 μg/mL of PEI and 1 μg/mL of CXCR4 plasmid (DNA/well). Similarly, CHO cells, stably expressing human CXCR2, were transfected with human WT GPR17, by the addition of a solution of PEI and GPR17 plasmid (1 μg/mL of DNA). Two days after transfection, the cells were used for experiments.

### 4.4. Co-Immunoprecipitation-Western Blot Assay

To verify the formation of CXCR2::GPR17 heterodimers, a co-immunoprecipitation assay was performed. CHO cells, expressing CXCR2 and transiently transfected with WT GPR17, were treated with Cangrelor (1 nM), Asinex 1 (5 nM), SB225002 (1 μM) and IL-8 (10 nM), alone or in combination, for 30 min. After the treatment period, cells were lysed and incubated overnight at 4 °C with an anti-GPR17 antibody (sc-74792, Santa Cruz Biotechnology, Dallas, TX, USA). After that, protein A-sepharose was used to obtain the immunocomplexes, which were solved by 7.5%-SDS-PAGE. Subsequently, PVDF membranes were challenged with an anti-CXCR2 (B01P, Abnova, Taipei, Taipei, Taiwan) or anti-CXCR4 (Sigma Aldrich, Milan, Italy) or an anti-GPR17 antibody (input samples). A chemiluminescent substrate (ECL, Perkin Elmer, Waltham, MA, USA) detected the signals. To ascertain the specificity of GPR17 interaction with CXCR2 or CXCR4, 1321N1 GPR17-expressing cells were immunoprecipitated using anti-GPR17 antibody and then the immunoprecipitated samples were blotted using an anti-CXCR2 or anti-CXCR4 antibody. ImageJ Software (version 1.41; Bethesda, MD, USA) was employed to perform a semi quantitative analysis of immunoreactive bands. The densitometric analysis values gave arbitrary units that were set to 100 % for control untreated cells. The optical density of each band presented in Figure 1E was normalized on the quantity of total proteins loaded due to the stain-free technology (Bio-Rad, Milan, Italy) [69].

### 4.5. ELISA Assay

An immunoenzymatic assay was used to assess the physical interaction between CXCR2, CXCR4 and GPR17. For the detection of CXCR2::GPR17 interaction, CHO cells, expressing CXCR2 and GPR17 receptors, were seeded in Petri dishes, and challenged with Asinex 1 (5 nM), SB225002 (1 μM) and IL-8 (10 nM), alone or in combination, for 30 min. For the detection of CXCR4::GPR17 interaction, 1321N1 cells were treated with SDF-1 (100 ng/mL), Asinex 1 (5 nM) and Plerixafor (500 nM), alone or in combination, for 30 min. Finally, to ascertain the specificity of GPR17 interaction with CXCR2 or CXCR4, 1321N1 GPR17-expressing cells were challenged with saline, IL-8 or SB225002 (for CXCR2-GPR17 interaction) or with SDF-1 or Plerixafor for 30 min.

Then, cells were lysed and transferred in well pre-coated with an anti-GPR17 antibody (sc-74792, Santa Cruz Biotechnology) for 90 min [15,70]. After washes, each well was incubated with 1% BSA for 20 min to block non-specific sites and then, challenged for 2 h with an anti-CXCR2 (Abnova, B01P) or CXCR4 antibody (Sigma Aldrich, Milan, Italy). Following extensive washes, an anti-rabbit HRP-conjugated antibody was added to each well for 2 h. The TMB substrate (Thermo Fisher Scientific, Waltham, Massachusetts, USA) was added, and the absorbance was read at 450 nm. Blanks were obtained by incubating the samples in the absence of the primary antibody. The home-made immunoenzymatic assay was set-up in previous experiments [70,71]. Moreover, primary antibodies specific for CXCR2 or CXCR4 did not evidence specific signals (Abs lower than 0.1 at 450 nm) on cell lysates. 

### 4.6. cAMP Assay

The functional interaction between CXCR2, CXCR4 and GPR17 was investigated by detecting the functional response of each receptor, i.e., by measuring the amount of intracellular cAMP upon receptor stimulation with the respective ligand. To this purpose, a competitive protein binding method was used following the procedure previously described [15]. Cells were seeded in 24-well plate and incubated with receptor agonist or antagonist for different times (5–120 min). After the incubation period, the cAMP radioligand binding assay was performed [15,17]. Briefly, cells were challenged with phosphodiesterase inhibitor Ro20-1724 (20 μM) and then with 10 μM Forskolin (FK) in the presence or absence of receptors agonist or antagonist for 15 min. After incubation, cells were lysed and cAMP was determined by using a radioligand binding assay [8]. Radioactivity was measured by liquid scintillation spectrometry.

### 4.7. Data Analysis

One-way ANOVA study followed by the Bonferroni test for repeated measurements was used for statistical analysis. Differences were considered statistically significant with *p* < 0.05.

### 4.8. Homology Modeling Procedures

A homology modeling approach was used to build the 3D structure of GPR17, since the experimental one is missing. The modeling was based on a multiple sequence alignment of a subclass of structurally related class-A GPCRs, as already described in our previous works [18,24]. The multiple sequence alignment was performed using the TM-Coffee algorithm, a module of the T-Coffee package optimized for transmembrane proteins [72]. Based on this alignment, both P2Y1 and CysLT1 show a very similar sequence identity with GPR17 (29.6% and 30.3%, respectively) and are phylogenetically related to this receptor [73]. So, they are both potentially good templates for modeling. However, in order to apply a conservative approach with respect to our most recent work [66], the X-ray structure of P2Y1 (PDB ID: 4XNW) [74] was chosen as template to model GPR17. A homology model was produced also for CXCR4 and CXCR2 on their own X-ray structure (PDB codes: 3ODU and 6LFL, respectively) [38,75] in order to fix any mutated residue, obtaining a representative structure of the wild type receptor. Since in all templates the third intracellular loop (ICL3) that connects TM5 and TM6 (res. 241–244 (6LFL), res. 229–230 (3ODU), res. 248–254 (4XNW)) was engineered, the missing residues in this region and all the other missing loops in the structure were rebuilt ab initio and refined by the “Loop refinement” program included in Prime (Schrödinger Release 2020-4: Prime, Schrödinger, LLC, New York, NY, USA, 2020) [76,77]. The implicit membrane refinement was used by importing coordinates from 3ODU.pdb [38] generated by OPM server [78] and an extended serial loop sampling was performed. The crystalized CXCR4::CXCR4 homodimer was equilibrated by a molecular dynamics (MD) simulation 250 ns long (see next paragraph for further description) and the equilibrated structure was used to generate homo/heterodimeric complexes after structural superposition of monomers on the CXCR4::CXCR4 homodimer, chosen as template also for the physiological relevance of its dimerization interface [61,62]. All the modeling procedures were carried out with the Schrödinger 2020-4 “Homology model” tool using the OPLS3e force field [79].

### 4.9. Molecular Dynamics Simulations

For the six dimers, namely CXCR2::CXCR2, CXCR2::CXCR4, CXCR2::GPR17, CXCR4::CXCR4, CXCR4::GPR17, GPR17::GPR17 and the three monomers, namely CXCR2, CXCR4 and GPR17, modeled as previously described, MD simulations 250 ns long were carried out in replicates (3 replicas for each system), by using the Desmond Molecular Dynamics System [80] (D. E. Shaw Research, New York, NY, USA, 2020-4; Maestro-Desmond Interoperability Tools, Schrödinger, New York, NY, USA, 2021). To avoid biases due to the high fluctuation of the unstructured C-terminus and to reduce the box dimensions, all the models were truncated at the end of transmembrane (TM) helix 7. The structures were then prepared and optimized for simulation by the Protein preparation wizard tool included in Maestro (Schrödinger Release 2020-4: Maestro, Schrödinger, LLC, New York, NY, USA, 2021) to assign the correct hydrogen atom topology, adjust protonation states, assign caps to N- and C-terminus (acetyl (ACE) and N-methyl amide (NME) groups, respectively) and minimize the systems according to the OPLS3e force field [79]. Then, by the Desmond System Builder tool, proteins were positioned into a POPC (1-palmitoyl-2-oleoyl-sn-glycero-3-phosphocholine) bilayer membrane after their orientation along the *Z*-axis performed by the OPM server [78], that allows spatial arrangements of membrane proteins with respect to the hydrocarbon core of the lipid bilayer. The dimerization interface was determined based on the spatial coordinates included in the X-ray structure of CXCR4 homodimer [38]. Specifically, a structural superposition of GPR17 and CXCR2 monomers on CXCR4 homodimer was performed to generate the homo- and hetero-dimers. Periodic boxes were planned by allowing at least 15 Å in the membrane plan and at least 10 Å in the perpendicular direction further than the peptide dimensions. Solvation was made with the TIP3P water model, adding NaCl at a concentration of 0.15 M plus the required counterions to neutralize system charge. Before running the production phase of MD simulations, the systems were relaxed according to the standard Desmond protocol for systems with membrane (see Supplementary Methods). MD production phase was performed for all the systems using the NPγT ensemble with Nosé-Hoover thermostat at 300 K, Martyina-Tobias-Klein barostat at 1.01325 bar and surface tension at 0 bar∙Å with semi-isotropic coupling. The OPLS3e force field was used with a cutoff for the coulombic interactions at 9 Å. The RESPA time integrator was used with time steps of 2 fs for bonded and near interactions and 6 fs for far interactions, for a total of 250 ns recording frames every 100 ps.

Before analysis, trajectories were sub-sampled every 500 ps and for cluster analysis the three replicas were merged in one trajectory. RMSD and fluctuation RMSF of C-alpha atoms, interactions (hydrogen bonds, salt bridges, cation-π and π-π) analysis, and cluster analysis were performed with three in-house made Python scripts leveraging the packages “topo”, “traj_util”, “traj”, and “analysis” of the “schrodinger.application.desmond.packages” library. While the first two scripts are straightforward application of the packages, cluster analysis was implemented according to the Gromos algorithm [81] and clusters were generated according to a RMSD threshold of 1.6 Å. The analyses of interactions between monomers were performed excluding the first 250 ns, considered as an equilibration step.

### 4.10. Dimer Formation Energy Calculation

The dimer formation energy was computed considering for each dimer three trajectories: the dimer itself and the two constituting monomers (or one monomer counted twice in case of the homodimers). For each aminoacidic chain of these systems the Schrödinger standard script “trj_interactions.py” was used to compute the following interaction energies: chain-membrane, chain-water, chain-ions and in case of a dimeric system chain-chain. The same script was used to compute the internal energy of each chain to keep account of energy associated to chain rearrangement. We define “dimer formation energy” as the sum of all these energy (chain-chain interaction counted only once) in the dimer simulation minus the sum of all these energies for the chain in monomer simulations. This represents the energy balance of the transition from two separate monomers to the dimer configuration and when negative indicates a favorable process. For this calculation, in the case of dimers simulations, only the last 250 ns of the trajectories were considered. This choice was made considering that, since they interact only with the membrane and the solvent, the energies of monomers are constant for the whole simulated time, while the interaction energy of dimers needs more time to equilibrate. Moreover, in this way, the statistics of the two energies has been computed on the same number of frames, making the results more comparable.

### 4.11. Plotting Procedures

All plotting was realized by leveraging Python matplotlib [82]. Interaction plots, in addition to scatterplots, show also a LOWESS nonparametric interpolation made using the statsmodels library [83].

## Data Availability

All data are contained within the manuscript.

## Supplementary Materials

The following supporting information can be downloaded at: https://www.mdpi.com/article/10.3390/ijms24010261/s1.

## Abbreviations

Chemokine receptor, CXCR; Forskolin, FK; G protein-coupled receptor, GPCR; molecular dynamics, MD; homology modelling, HM; root mean square deviation, RMSD; root mean square fluctuation, RMSF.
