# Supplementary material for: Functional Heterodimerization between the G Protein-Coupled Receptor GPR17 and the Chemokine Receptors 2 and 4: New Evidence
Source: Int J Mol Sci. 2022 Dec 23;24(1):261. doi: 10.3390/ijms24010261 (PMC9820414; doi:10.3390/ijms24010261)
Supplement: Supplementary file 1 [file ijms-24-00261-s001.zip › ijms-1971690-supplementary.pdf]

Supplementary Material

Functional Heterodimerization between the G Protein-Coupled Receptor GPR17 and the Chemokine Receptors 2 and 4: New Evidence

Simona Daniele [1,†], Simona Saporiti [2,†], Stefano Capaldi [3], Deborah Pietrobono [1], Lara Russo [1], Uliano Guerrini [2], Tommaso Laurenzi [2], Elham Ataie Kachoie [3], Luca Palazzolo [2], Vincenzo Russo [4], Maria Pia Abbracchio [5], Ivano Eberini [6,*] and Maria Letizia Trincavelli [1]

[1] Dipartimento di Farmacia, Università di Pisa, Via Bonanno 6, 56126 Pisa, Italy; simona.daniele@unipi.it (S.D.); deborah.pietrobono@farm.unipi.it (D.P.); lara.russo@phd.unipi.it (L.R.); maria.trincavelli@unipi.it (M.L.T.)

[2] Dipartimento di Scienze Farmacologiche e Biomolecolari, Università degli Studi di Milano, Via Balzaretti 9, 20133 Milan, Italy; simona.saporiti@unimi.it (S.S.); uliano.guerrini@unimi.it (U.G.); tommaso.laurenzi@unimi.it (T.L.); luca.palazzolo@unimi.it (L.P.)

[3] Dipartimento di Biotecnologie, Università degli Studi di Verona, Strada Le Grazie 15, 37134 Verona, Italy; stefano.capaldi@univr.it (S.C.); elham.ataiekachoie@univr.it (E.A.K.)

[4] Cancer Gene Therapy Unit, Program of Immunology and Bio Immuno Gene Therapy of Cancer, Division of Molecular Oncology Scientific, Institute San Raffaele, 20132 Milan, Italy; russo.vincenzo@hsr.it

[5] Laboratorio di Farmacologia Molecolare e Cellulare Della Trasmissione Purinergica, Dipartimento di Scienze Farmaceutiche, Università Degli Studi di Milano, Via Balzaretti 9, 20133 Milan, Italy; mariapia.abbracchio@unimi.it

[6] Dipartimento di Scienze Farmacologiche e Biomolecolari & Data Science Research Center (DSRC), Università degli Studi di Milano, Via Balzaretti 9, 20133 Milan, Italy

* Correspondence: ivano.eberini@unimi.it

† These authors contributed equally to this work.

Supplementary Methods

Protocol for relaxation of a membrane-containing system

The protocol is carried out in six stages, organized as follow:

1. NVT ensemble, brownian dynamics, 100 ps at 10 K with a protein restrain of 50 kcal mol^{-1}Å^{2}.

2. NPT ensemble, brownian dynamics, 20 ps at 100 K and 1000 bar with a membrane heavy atoms z restrain of 5 kcal mol^{-1}Å^{-2} and protein heavy atoms restrain of 20 kcal mol^{-1}Å^{-2}.

3. NPγT ensemble, 100 ps at 100 K and 1000 bar with a membrane P and N atoms z restrain of 2 kcal mol^{-1}Å^{-2} and protein heavy atoms restrain of 10 kcal mol^{-1}Å^{-2}.

4. NPγT ensemble, 150 ps heating from 100 K to 300 K at 100 bars with a membrane P and N atoms z restrain of 2 kcal mol^{-1}Å^{-2} and protein heavy atoms restrain of 10 kcal mol^{-1}Å^{-2}. The restrains were gradually reduced to 0.

5. NVT ensemble, 50 ps at 300 K and backbone heavy atoms restrain of 50 kcal $mol^{-1}Å^{-2}$.

6. NVT ensemble, 50 ps at 300 K, no restrains.

All the steps are run with Nosé-Hoover thermostat and Martyna-Tobias-Klein barostat. In stages 2-4 a gaussian biasing force is applied in order to prevent water from permeating the membrane.

Supplementary Figures

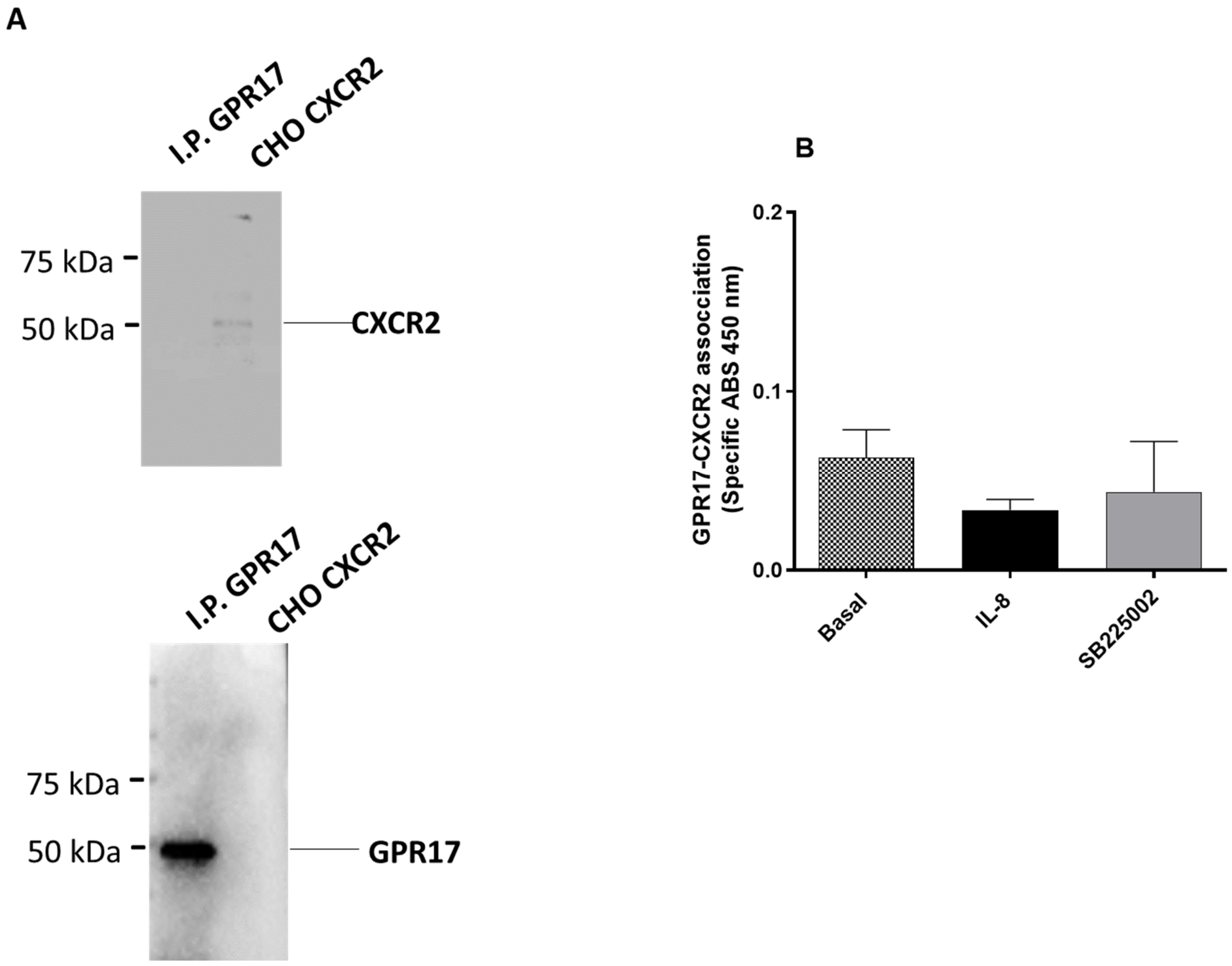

Figure S1. Specificity of CXCR2-GPR17 interaction evaluated by Western Blot/co-immunoprecipitation and immunoenzymatic experiments. (A) 1321N1 cells stably transfected with GPR17, were lysed and immunoprecipitated with an anti-GPR17 antibody and then subjected to western blot analysis using a specific antibody for CXCR2 (upper panel) or GPR17 (bottom panel). Lysates (30 µg) from CHO CXCR2-expressing cells (i.e., stably transfected) were used as positive control for the CXCR2 antibody. A representative western blot is shown. (B) 1321N1 cells stably transfected with GPR17 were treated with saline, IL-8 or SB225002 for 30 min. Following treatments, equal amounts of cell lysates (30 µg) were captured on wells pre-coated with GPR17 antibody. After extensive washing, the levels of the GPR17-CXCR2 complex were quantified using an antibody

specific for CXCR2 by immunoenzymatic assay, as reported in the Methods section. Blanks were obtained in the absence of the primary antibody. The data are reported as specific absorbance at 450 nm.

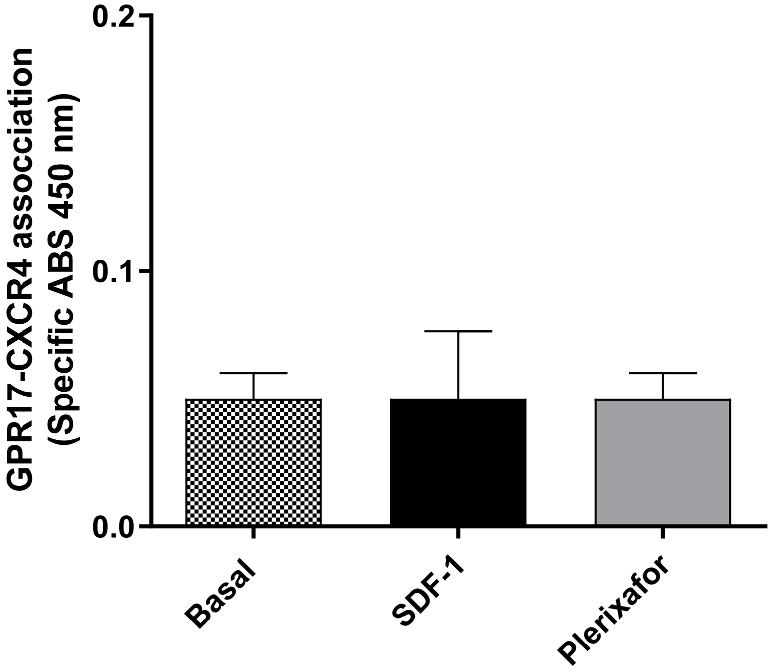

Figure S2. Specificity of CXCR4 and GPR17 interaction by immunoenzymatic experiments. 1321N1, cells stably transfected with GPR17, and not expressing CXCR4 receptors, were treated with saline, SDF-1 or Plerixafor for 30 minutes. Following treatments, equal amounts of cell lysates were captured on wells pre-coated with GPR17 antibody. After extensive washing, the levels of the GPR17-CXCR4 complex were quantified using an antibody specific for CXCR4 by a specific immunoenzymatic assay. Blanks were obtained in the absence of the primary antibody. The data are reported as specific absorbance at 450 nm.

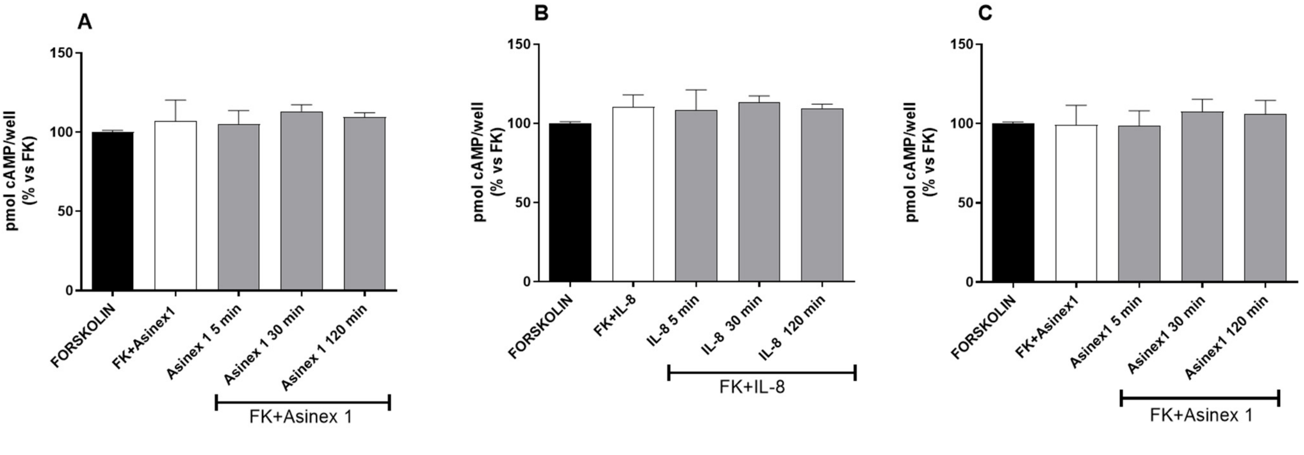

Figure S3. Specificity of CXCR2, CXCR4 and GPR17 in desensitization experiments. (A) CHO cells, stably expressing CXCR2, were treated for different times (5-120 min) with Asinex 1, washed, and then stimulated with FK in the presence of Asinex 1. (B) 1321N1 cells, stably expressing GPR17, were treated for different times (5-120 min) with IL-8, washed, and then stimulated with FK in the

presence of IL-8. (C) 1321N1 cells were transiently transfected with CXCR4, and treated for different times (5-120 min) with Asinex 1, washed, and then stimulated with FK in the presence of Asinex 1. cAMP quantification was assessed *via* a radioligand assay in lysates of these cells. The data are expressed as percentage of pmol cAMP/well versus FK. The significance of the differences was determined with a one-way ANOVA with Bonferroni post-test. The significance of the differences was determined with a one-way ANOVA with Bonferroni post-test.

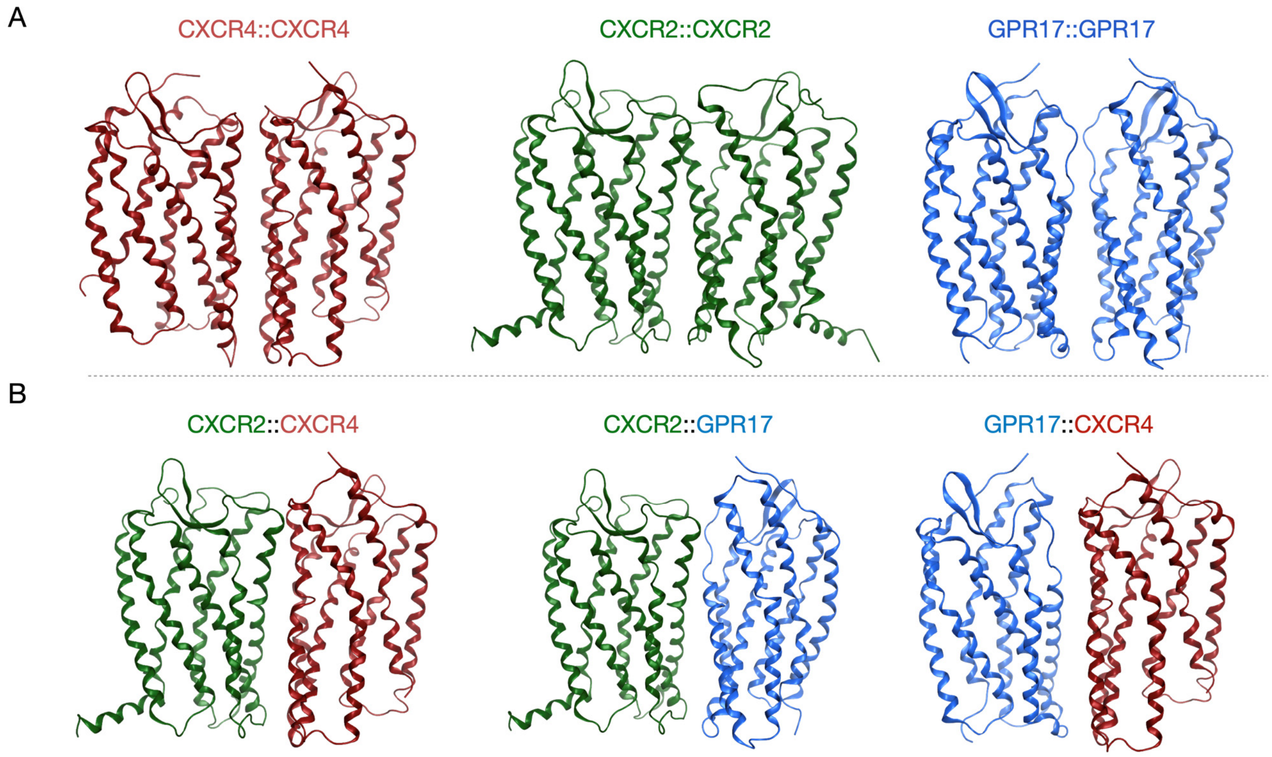

Figure S4: Structural representation of homo/heterodimers. (A) CXCR4::CXCR4, CXCR2::CXCR2, GPR17::GPR17 homodimers; (B) CXCR2::CXCR4, CXCR2::GPR17 and GPR17::CXCR4 heterodimers. Secondary structures are rendered as ribbons colored according to the specific receptor color code.

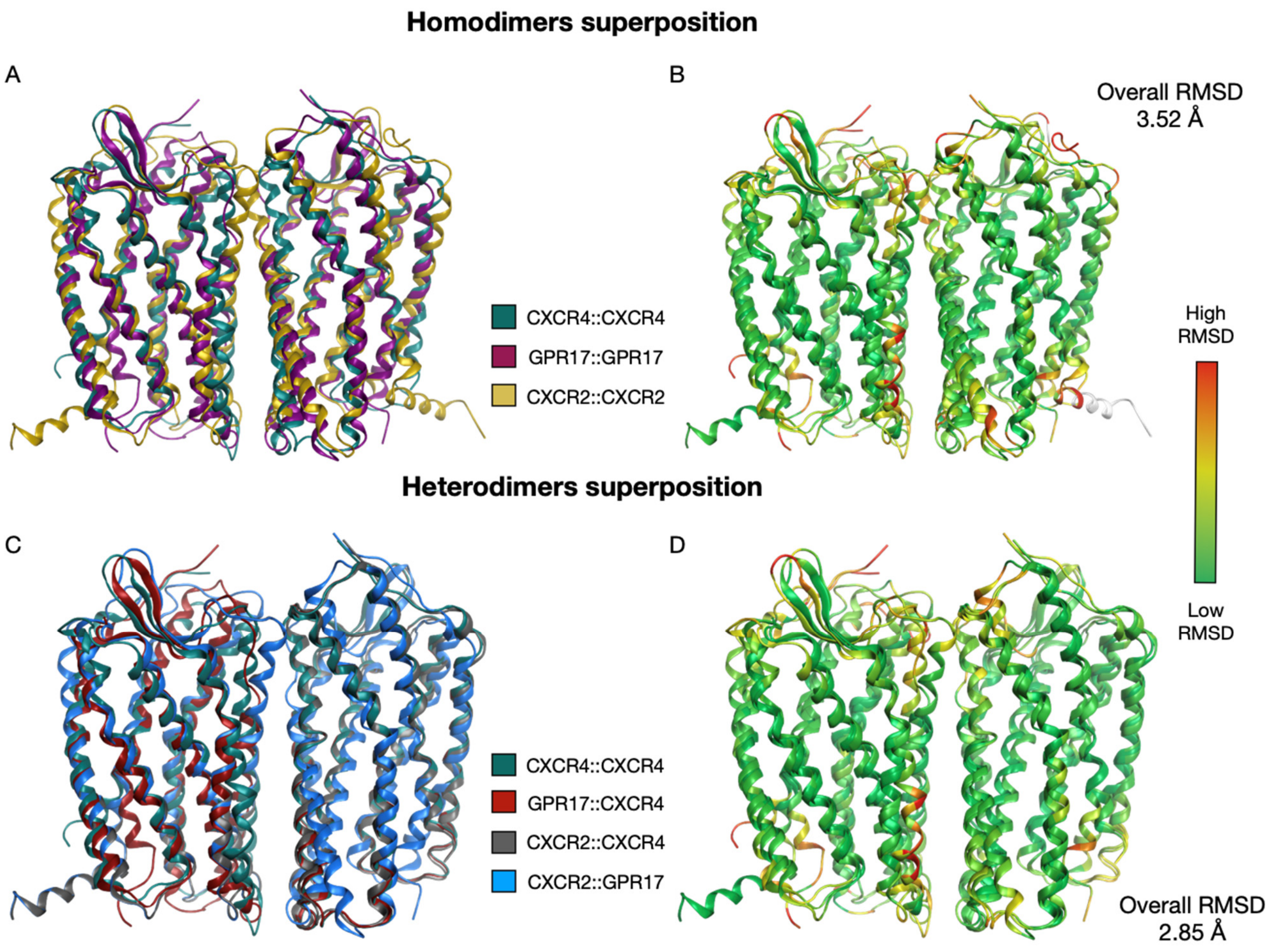

Figure S5: Structural comparison between the crystalized CXCR4 homodimer and all the modeled dimers. Structural superposition between the X-ray structure of CXCR4 homodimer (PDB ID: 3ODU) with homo- (A,B) and hetero-dimeric (C,D) complexes. The structures are represented as ribbons and colored by complex, on the left, or by RMSD, on the right.

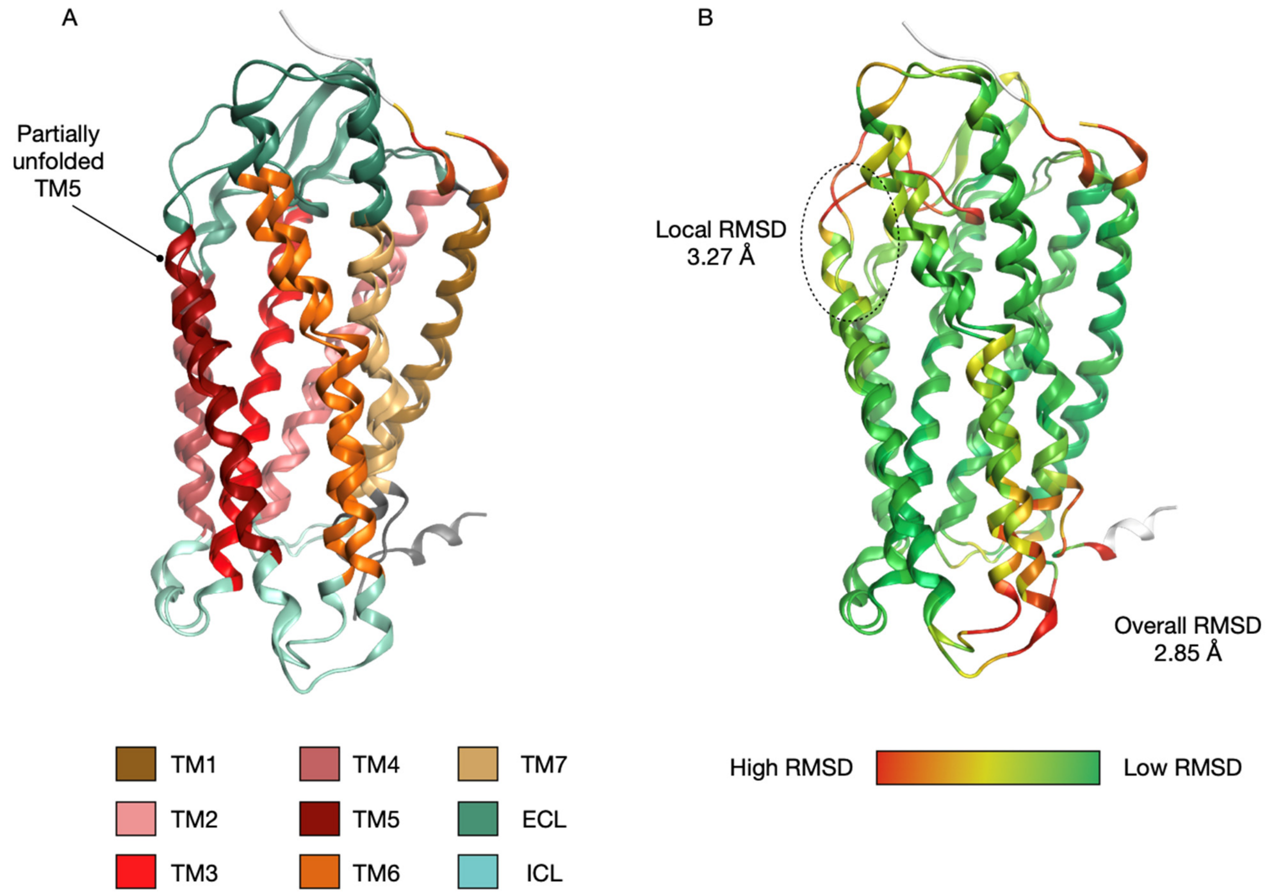

Figure S6: Structural comparison between GPR17 model and Cryo-EM structure. Structural superposition between GPR17 homology model and the experimentally solved GPR17 structure (PDB ID: 7Y89) represented as ribbons colored according to MOE GPCR annotation (A) or RMSD gradient (B) with the local and global RMSD values indicated in the picture.

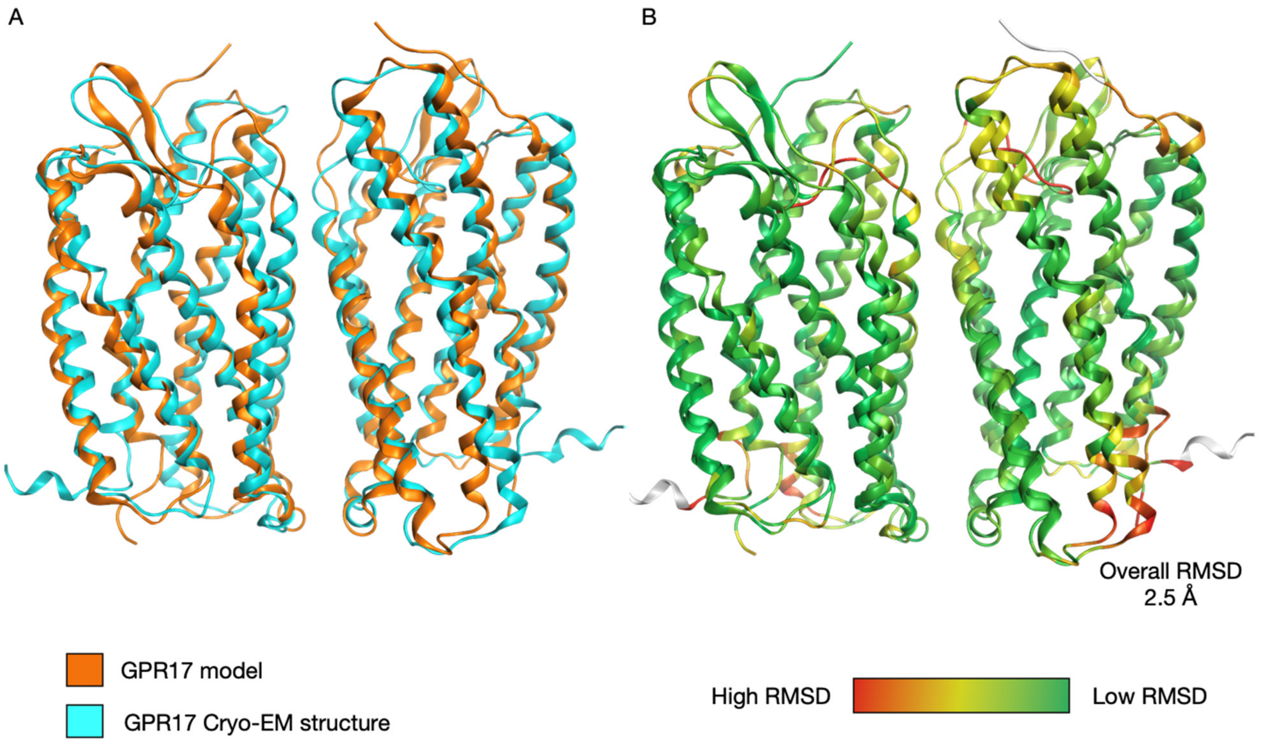

Figure S7: Structural comparison between the model of GPR17 homodimer and Cryo-EM structure. Comparison between the homology model of GPR17 homodimer and a GPR17 homodimer obtained by superposition of the (duplicated) Cryo-EM structure (PDB ID: 7Y89) with the model. Structures are represented as ribbons and colored by complex (A) or by RMSD (B).

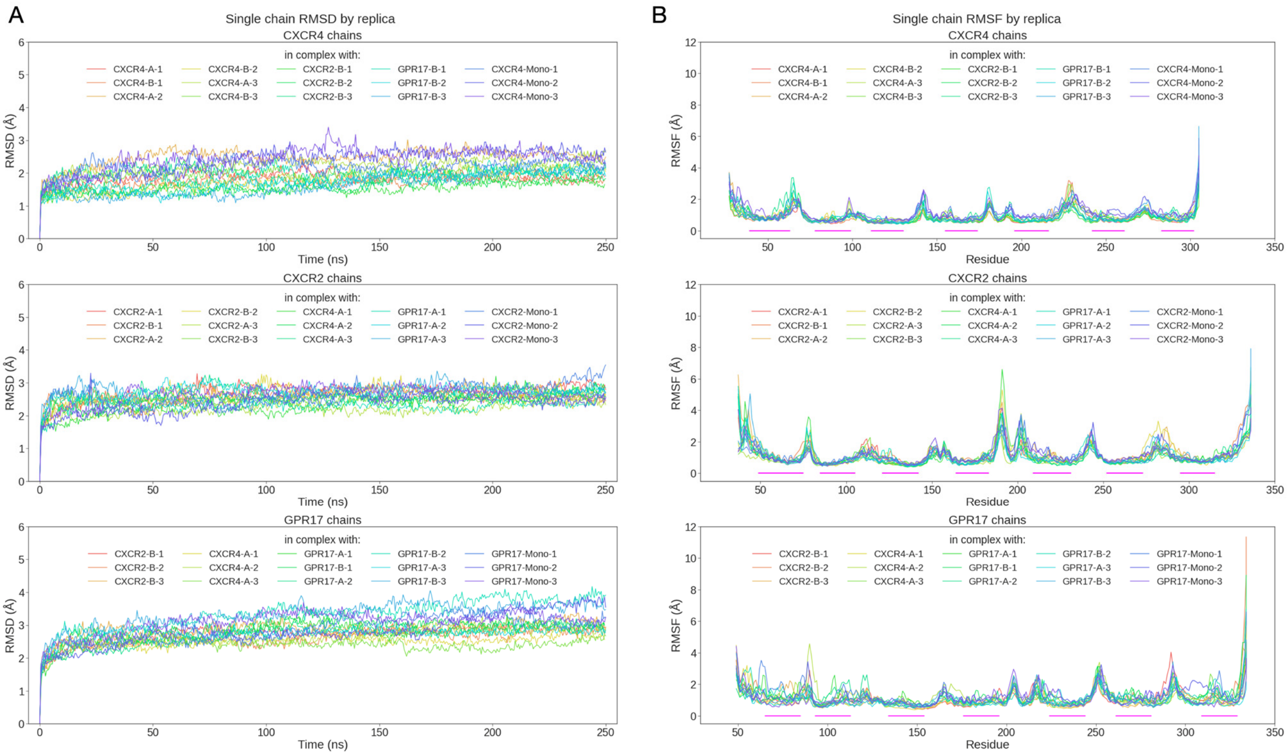

Figure S8: Single chain RMSD and RMSF by replica. (A) RMSD computed for C-alpha atoms of CXCR4, CXCR2 and GPR17 in homo/heterodimeric complex and in monomeric form. According to this analysis all the chains stabilize within the first 20 ns of simulation. (B) RMSF computed for C-

alpha atoms of CXCR4, CXCR2 and GPR17 in homo/heterodimeric complex and in monomeric form. Lines in magenta indicate the residues included in α-helix transmembrane domains. The major fluctuations have been identified for loop regions. In all the plots, the legend reports the name of the chain to which the plotted chain is complexed in the dimer or the indication of monomeric state.

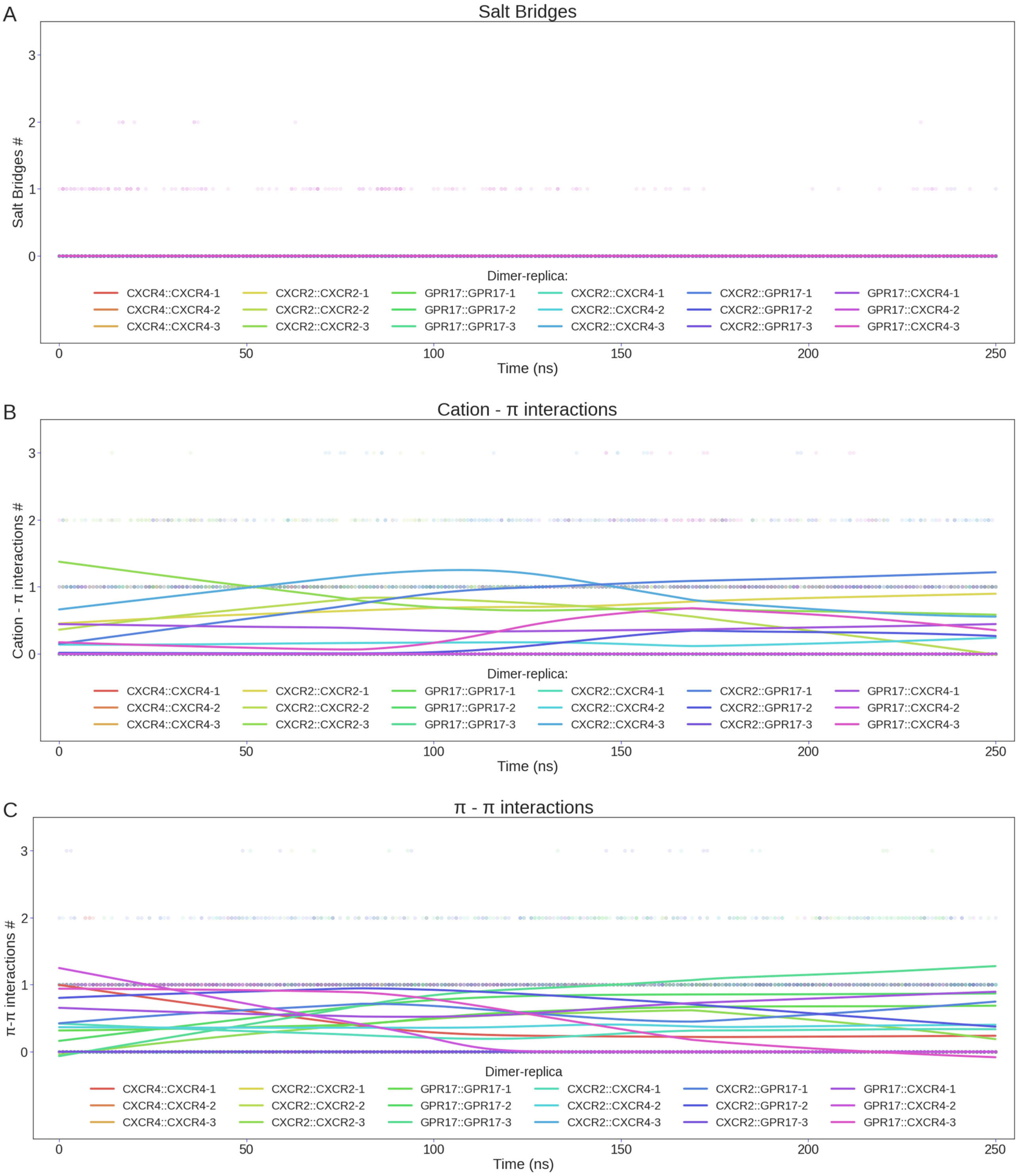

Figure S9: Scatter plots of salt bridges, π-π and Cation-π interactions by replica. (A) Salt bridges number vs simulation time. One interaction, even if not conserved during time, has been observed in the GPR17::CXCR4 system. (B) π-π interactions number vs simulation time; all the systems show a number of π-π in the range between 0 and 1. (C) Cation-π interactions vs simulation time. Also in

this case the number of bonds is approx. between 0 and 1. This data suggests the lower impact of these interaction types on the dimerization process. In addition to the scatterplot a LOWESS nonparametric interpolation is shown.

Table S1: H-bonds interactions in homodimers. Reported bonds occur with a frequency threshold of 20%; residues are numbered according to the Ballesteros-Weinstein scheme.

CXCR2::CXCR2		CXCR4::CXCR4		GPR17::GPR17	
**Chain A**	**Chain B**	**Chain A**	**Chain B**	**Chain A**	**Chain B**
Val$^{3.55}$	Lys$^{4.38}$	Tyr$^{3.51}$	Pro$^{34.56}$	His$^{5.36}$	Tyr$^{5.36}$
His$^{3.56}$	His$^{3.56}$, Thr$^{34.51}$, Leu$^{34.54}$	Ala$^{34.50}$, Thr$^{34.51}$, Asn$^{34.52}$	Asn$^{34.52}$	Lys$^{34.52}$	His$^{3.56}$
Thr$^{34.51}$	Thr$^{34.51}$	Arg$^{34.55}$	Val$^{3.55}$	Lys$^{34.55}$	Val$^{3.55}$
Leu$^{34.54}$, Lys$^{34.57}$	His$^{3.56}$	Pro$^{34.56}$	Tyr$^{3.51}$	Leu$^{4.40}$	Arg$^{5.63}$
Leu$^{4.41}$	Tyr$^{3.51}$	Asn$^{5.32}$	Glu$^{6.64}$	Tyr$^{4.41}$	Ser$^{5.64}$
Asn$^{5.34}$, Trp$^{5.35}$	Thr$^{6.63}$	Leu$^{6.63}$	Asn$^{5.32}$, Trp$^{5.35}$	His$^{5.38}$	His$^{5.38}$
Ser$^{5.45}$	Ser$^{5.45}$, Gly$^{5.461}$	Glu$^{6.64}$	Asn$^{5.32}$	Arg$^{5.63}$	Tyr$^{4.41}$
Thr$^{6.59}$, Arg$^{6.62}$	Asn$^{5.34}$				

Table S2: H-bonds interactions in heterodimers. Reported bonds occur with a frequency threshold of 20%; residues are numbered according to the Ballesteros-Weinstein scheme.

CXCR2::CXCR4		CXCR2::GPR17		GPR17::CXCR4	
**CXCR2**	**CXCR4**	**CXCR2**	**GPR17**	**GPR17**	**CXCR4**
Tyr$^{3.51}$	Pro$^{34.56}$, Leu$^{4.39}$	Tyr$^{3.51}$, Val$^{3.55}$, His$^{3.56}$	Lys$^{34.55}$	Val$^{34.51,}$ Lys$^{34.52}$	Asn$^{34.52}$
Ala$^{34.50}$	Arg$^{34.55}$	Arg$^{34.52}$	Lys$^{34.52}$	Lys$^{34.55}$	His$^{3.56}$, Asn$^{34.52}$
Thr$^{34.51}$	Asn$^{34.52}$	Thr$^{34.53}$	His$^{3.56}$	Leu$^{5.41}$	Gln$^{5.42}$
Thr$^{34.53}$, Leu$^{34.54}$	His$^{3.56}$	Lys$^{34.57}$	Val$^{3.55}$	Arg$^{6.63}$	Phe$^{45.53}$
Lys$^{34.57}$	Val$^{3.55}$, Ser$^{5.63}$, His$^{5.67}$	Tyr$^{34.59}$	Ser$^{5.64}$		
Ser$^{4.48}$	Ser$^{5.56}$	Ser$^{4.48}$	Thr$^{5.56}$		
Thr$^{6.63}$, Gln$^{6.64}$	Asn$^{5.32}$, Trp$^{5.35}$	Asn$^{5.34}$	His$^{5.39}$, Tyr$^{6.62}$, Ser$^{6.64}$, Val$^{6.59}$		
		Ser$^{5.45}$	Leu$^{5.41}$		
		Thr$^{6.63}$	Ser$^{5.37}$, Ser$^{6.64}$		
		Gln$^{6.64}$	His$^{5.38}$		
